# Gender and ethnicity bias in medicine: a text analysis of 1.8 million critical care records

**DOI:** 10.1093/pnasnexus/pgac157

**Published:** 2022-08-18

**Authors:** David M Markowitz

**Affiliations:** School of Journalism and Communication, University of Oregon, Eugene, OR, USA

**Keywords:** bias, gender, ethnicity, language, medicine

## Abstract

Gender and ethnicity biases are pervasive across many societal domains including politics, employment, and medicine. Such biases will facilitate inequalities until they are revealed and mitigated at scale. To this end, over 1.8 million caregiver notes (502 million words) from a large US hospital were evaluated with natural language processing techniques in search of gender and ethnicity bias indicators. Consistent with nonlinguistic evidence of bias in medicine, physicians focused more on the emotions of women compared to men and focused more on the scientific and bodily diagnoses of men compared to women. Content patterns were relatively consistent across genders. Physicians also attended to fewer emotions for Black/African and Asian patients compared to White patients, and physicians demonstrated the greatest need to work through diagnoses for Black/African women compared to other patients. Content disparities were clearer across ethnicities, as physicians focused less on the *pain* of Black/African and Asian patients compared to White patients in their critical care notes. This research provides evidence of gender and ethnicity biases in medicine as communicated by physicians in the field and requires the critical examination of institutions that perpetuate bias in social systems.

Significance Statement Bias manifests in many social systems, including education, policing, and politics. Gender and ethnicity biases are also common in medicine, though empirical investigations are often limited to small-scale, qualitative work that fails to leverage data from actual patient–physician records. The current research evaluated over 1.8 million caregiver notes and observed patterns of gender and ethnicity bias in language. In these notes, physicians focused more on the emotions of women compared to men, and physicians focused less on the emotions of Black/African patients compared to White patients. These patterns are consistent with other work investigating bias in medicine, though this study is among the first to document such disparities at the language level and at a massive scale.

Bias in medicine is well-documented in the patient–physician relationship. Men, for example, are often treated more comprehensively than women for a range of symptoms (1). Patients with chronic pain are also viewed differently based on their gender, as women are often perceived as hysterical and emotional compared to men, who are often perceived as brave and strong (2). Medical inequities are systematic by ethnicity as well (3). Black patients are 40% less likely to receive standard cardiac catheterization treatment compared to White patients (4), and in other settings (e.g. pain management and surgical safety), ethnic minorities are provided inadequate treatment compared to White patients (5–7). The marginalization of women and ethnic minorities is widespread in medicine, yet few studies can document these patterns at scale using actual patient–physician records.

Recent work has addressed this opportunity by collecting physician notes from 600 medical cases and analyzing how physicians describe patients based on demographics. Physicians cast more judgment or doubt when communicating with Black patients than with White patients, but no differences emerged by gender (8). Other evidence suggests physicians with high rates of implicit racial bias toward Black patients tend to use more anxiety words in their interactions compared to physicians with low rates of implicit racial bias (9). These studies provide some of the first data suggesting how language plays a central role in the patient–physician relationship from medical records and patient interactions. However, the constrained samples and limited number of linguistic features examined presents a challenge to identify how bias might manifest in medicine as an institution. The current work draws on these empirical foundations to evaluate how bias and linguistic disparities are reflected in word patterns of physicians at a massive scale for a single hospital. Caregiver notes from over 1.8 million patient–physician records were assessed to understand what physicians attended to as they wrote about women and ethnic minorities compared to men and White patients.

## The psychology of language: content and style words

In the social sciences, language patterns are often used as indicators of one’s psychological focus (10, 11). This *words-as-attention* approach to text analysis has been substantiated in hundreds of studies (12–14), with the main thesis suggesting researchers can glean psychological information about communicators by simply counting their words (15). For example, prior work has used language to identify where people of high or low social status focus their attention. Leaders, or people of high social rank, tend to focus more on the collective as revealed by their greater use of “we” words compared to followers, or people of low social rank (16). Other work suggests language connects to personality (17) and how people communicate about groups they also dehumanize (18, 19). Together, words matter, and they are instrumental to understand the internal processing of communicators.

What types of word patterns reveal one’s psychological focus? In general, two classes of words are often investigated: content words and style words. Content words (e.g. nouns and verbs) describe *what* people are talking about and style words (e.g. pronouns, articles, and prepositions) describe *how* people are communicating (10). Style words are often connected to a range of social and psychological processes, including the marginalization of (out)groups. In a related study, Markowitz and Slovic (18) had participants rate the humanity of immigrants on an evolution scale and then describe their feelings toward them. Those who dehumanized (e.g. rated immigrants as less evolved) tended to use more impersonal pronouns to describe them (e.g. words such as *it, anyone*, and*someone*) compared to those who rated immigrants as more evolved. Research on bias in law enforcement also suggests police officers speak less respectfully to Black residents compared to White residents during Oakland, CA traffic stops (20). Together, marginalized groups in society (e.g. women and minorities) are often undermined in many settings and language patterns can reveal characteristics of psychological processes like bias via naturally occurring data (21, 22). The current work uses large-scale text analyses and an automated approach to document bias-based disparities in medicine.

### Predictions

Bias is expected to manifest in ways that are consistent with prior evidence. First, physicians will focus more on emotions and the emotional state of women compared to men (H_1_) because women are often perceived as more hysterical, emotional, and dramatic in medical settings than men (2, 23). The perception that women are more emotional than men is a stereotype in medicine (24). For example, surveyed medical professionals believe women are more likely to report pain (25) and exaggerate their negative experiences than men (26, 27). The stereotype that women are “just more emotional” than men can have downstream consequences for their care. That is, physicians who focus more on a patient’s emotions and their emotional experiences may provide them with inequitable or inadequate treatment (e.g. over-prescribing or under-prescribing medication) (28), believing that a woman’s condition is psychological and exaggerated, not biological nor physical. This first hypothesis, therefore, investigates how linguistic disparities related to emotion operate across patient genders.

By focusing more on emotions and emotional experiences for women compared to men, physicians cannot fully attend to other aspects of a patient that might be instrumental to their health (e.g. medical diagnoses and physical aspects of the patient). A second prediction offers a complementary hypothesis to H_1_ and suggests physicians will focus more on the bodily diagnoses of men compared to women (H_2_). This prediction is conceptually consistent with research on gender bias in other settings (2, 8), where journalists who ask questions to tennis players who are men tend to focus more on the match compared to journalists who ask questions to tennis players who are women (29). Questions to tennis players who are men included words such as *clay, tie, sets*, and *serve*, whereas questions to tennis players who are women included words such as *nervous, mom, improve*, and *father*. Therefore, bias can be revealed by how much emotion (H_1_) or context-relevant terms (e.g. references to the body in medicine; H_2_) are the psychological focus of people who communicate to different genders.

Bias can also appear in pronoun use to reflect one’s psychological distance toward a group. One category of pronouns, impersonal pronouns, relates to the current research because they describe how a physician may depersonalize a patient’s care and distance themselves from the patient psychologically. Prior work suggests men often receive more personalized time and attention than women from nurses or physicians (30, 31). Therefore, this social dynamic and disparity should also be reflected in the language of physicians via pronouns to indicate more personalized attention and focus provided to men versus women. H_3_ predicts physicians will use more impersonal pronouns to describe women than men as a reflection of the perceived psychological distance between a physician and their target (32–35).

In addition to such a priori predictions, two exploratory linguistic measures were used to indicate a physician’s cognitive thinking style when attending to different patients: analytic thinking and cognitive processing terms. Analytic thinking is a composite variable of style words that proxies Kahneman’s System 2 thinking (36–38). High scores on an analytic thinking index often reflect “cold” or “dispassionate,” but reasoned and structured communication (39). Alternatively, low scores on an analytic thinking index reflect a narrative or dynamic thinking style. Analytic thinking is a critical measure to understand cognitive styles of communicators and can indicate how physicians are being intentional and reasoned in their thinking across genders and ethnicities. Given the relatively uninvestigated link between bias, gender, ethnicity, and analytic thinking, these relationships were considered exploratory.

Cognitive processing terms describe a communicator “working through” an issue (39–41). People who use terms such as *although, should*, or *instead* are attempting to organize their thoughts and manage their appraisal of a target (42). In other words, cognitive processing terms describe how much people are questioning an unsettled issue or the amount of cognitive effort communicators are putting in to understand an unsettled task, which might be greater for groups that are marginalized because physicians are less certain about how they feel toward such groups (or, they have less experience attending to certain patients and need to put in more cognitive effort). Like the analytic thinking effect, it is unclear how much effort physicians might put into their patients as a reflection of their gender and ethnicity, or how structured their thinking process might be when attending to different groups of people. Therefore, this relationship is exploratory as well. Analytic thinking and cognitive processing are used in tandem to evaluate how language patterns can indicate the cognitive, organizational steps physicians went through psychologically to evaluate patients in critical care.

Note, while hypotheses are offered for gender disparities, these effects are also explored across ethnicities to indicate how physicians might attend to patients of different backgrounds as revealed by word patterns. Formal predictions by ethnicity are not offered because it is unclear how physicians may attend to emotions, physical aspects of one’s care, and personalize one’s health for different groups of non-White patients across diagnoses, though it is reasonable to expect linguistic disparities across ethnicities based on prior person–perception research (43). The stereotype content model, for example, suggests people often categorize racial and ethnic groups in systematic ways across warmth and competence dimensions: Asian people are often perceived as more competent than warm (44), in some cases African Americans are perceived as warm but incompetent (45), and Latinos are stereotypically low on warmth and competence dimensions (46). Here, the present research draws on principles of the stereotype content model by arguing that stereotypes are pervasive in person–perception, and such evaluations of others can facilitate disparities in medicine that are revealed in language. This work is one of the first studies to evaluate how such linguistic disparities manifest at scale.

Taken together, linguistic bias toward women and ethnic minorities was evaluated in 1.8 million caregiver notes. This research is critical because inequalities by gender and ethnicity are common in medicine, though they have rarely been documented in the linguistic reports by caregivers from patient medical charts (9, 47, 48). The goal of this research is to identify new linguistic pathways that indicate disparities in medicine and with this evidence, motivate change toward equity.

## Method

Deidentified patient medical records were obtained by the Medical Information Mart for Intensive Care (MIMIC-III) database (49–51). This archive contains medical details and descriptions for nearly 46,000 critical care patients and over 58,000 hospital admissions for individuals admitted to Beth Israel Deaconess Medical Center in Boston, MA. Hospital admissions include patients from 2001 to 2012 and each patient was tagged with demographic data (except for age), their diagnosis, prescribed medications, and vital signs. Caregiver notes were recorded by doctors, nurses, and other medical providers to describe the patients’ status, updates on their progress, and impressions from the caregiver. The current work used these notes to evaluate the psychological focus of physicians and identify patterns of bias.

Patients often received visits from multiple caregivers for the same hospital admission and the same person may revisit the hospital over time. Therefore, the entire archive contained a total of 1,851,281 patient–physician records with caregiver notes (502,221,132 words), after excluding those without text and those missing hospital admissions data (*n *= 231,899). The unit of analysis in this work is the individual caregiver note. On average, caregiver notes contained 271.28 words (*SD* = 383.12 words)^a^ and a breakdown of the sample by gender and ethnicity variables is offered in Table 1.

**Table 1.** Descriptive information across groups.

		Count	Percentage (%)
Gender	**Men**	**1,047,816**	**56.60**
	**Women**	**803,465**	**43.40**
Ethnicity	**Asian**	**55,249**	**2.98**
	Men	34,181	61.87
	Women	21,068	38.13
	**Black/African**	**180,410**	**9.75**
	Men	81,770	45.32
	Women	98,640	54.68
	**Hispanic or Latino**	**65,218**	**3.52**
	Men	40,149	61.56
	Women	25,069	38.44
	**White**	**1,295,941**	**70.00**
	Men	748,717	57.77
	Women	547,224	42.23
	**Other**	**254,463**	**13.75**
	Men	142,999	56.20
	Women	111,464	43.80

Note: percentages in bold were calculated by dividing the raw count by the total number of caregiver reports in the MIMIC-III database (*N *= 1,851,281). Unbolded percentages are calculated within each ethnicity group.

### Automated text analysis

All caregiver notes were processed by the automated text analysis tool, Linguistic Inquiry and Word Count (LIWC) (52). LIWC counts words as a percentage of the total word count per text and identifies a range of categories from its internal dictionary, including social dimensions (e.g. words related to family), psychological dimensions (e.g. words related to emotion), and part of speech dimensions (e.g. pronouns, articles, and prepositions). For example, the phrase “The patient is in bad health” contains six words and LIWC identifies the following words across its internal dictionary of categories, including but not limited to: articles (*the*; 16.67% of the total word count), negative emotion terms (*bad*; 16.67%), and health terms (*health*; 16.67%). LIWC is a gold-standard text analysis program for dictionary-based evaluations of language data; its dictionary and word counting approach have been validated in hundreds of studies (12, 13, 53, 54).

### Measures

A linguistic profile for each caregiver note was created using six language dimensions in the current study:

(1) and (2) Positive emotion terms (e.g. *brave, safe*, and *gentle*) and negative emotion terms (e.g. *bad, weak*, and*panic*) to evaluate a physician’s focus on a patient’s emotional state and their emotional experience (H_1_);
(3) Body terms (e.g. *nerve, spine*, and *stomach*) to assess how much a physician focuses on biological and physical aspects of a patient (H_2_);
(4) Impersonal pronouns (e.g. *it, someone*, and*who*) to indicate a physician’s perceived psychological distance to a patient through style words (H_3_);
(5) Analytic thinking^b^ to evaluate the cognitive thinking and reasoning style of a physician (exploratory) (55, 56); and
(6) Cognitive processing terms (e.g. *solve, determine*, and*perhaps*) to indicate how much effort a physician might expend to understand and work through issues related to a patient (exploratory).

All language dimensions were drawn from the standard LIWC2015 dictionary, and a correlation matrix of these variables is offered in Table S1.

## Results

### Analytic approach

This paper used linear mixed models to account for dependencies in the data. Random intercepts included the patient ID (to control for multiple hospital visits by the same patient), hospital stay ID (to control for multiple observations related to the same medical case), diagnosis (to control for baseline effects of how caregivers react to specific patient conditions), and physician ID (to control for baseline differences in physician writing styles).^c^ Simultaneous fixed effects included reported patient gender (Men or Women)^d^ and ethnicity (Asian, Black/African, Hispanic, Other, or White).^e^ Despite controlling for physician ID, physician gender and ethnicity were unavailable in the dataset and, therefore, not included in statistical models.^f^

Full linear mixed model outputs (Tables S2–S7) and multiple comparisons across language dimensions are available in the online supplement.

### Linguistic indicators of gender bias

The data revealed evidence of gender bias and disparities in the language of caregiver notes (see Table 2 for estimated marginal means and effect sizes across measures). Specifically, after controlling for patient ethnicity, physicians focused on more emotion for women compared to men. This effect was consistent across emotional valence, as physicians focused on more positive (*P *< 0.001) and negative emotion (*P *< 0.001) in their notes for women compared to men, supporting H_1_. To contextualize these results, consider the following excerpts from patients who had the same caregiver, a similar diagnosis, and were the same ethnicity (White), but different gender. The man received the following physician note, “S/P AVR with 5/10 incisional pain, grimacing with CDB and at rest,” which contains two negative emotion terms (*pain* and *grimacing*).^g^ The woman received the following physician note, “Extremely anxious, crying and becoming very worked up. Patient can not state what exactly the problem is but cries and exclaims help me and oh dear repetitively,” which contains four negative emotion terms (*anxious, crying, problem*, and *cries*). Another excerpt from the same caregiver toward a White woman suggested, “Pt extremely nervous and anxious throughout shift. Pt has fear that she is going to fall OOB and uneasy about all nursing care,” which contains four negative emotion terms (*nervous, anxious, fear*, and *uneasy*). In contrast, a man was described as “Pt pleasantly confused,” with one negative emotion term (*confused*), a note that is still generally positive.

**Table 2.** Estimated marginal means by gender across language dimensions.

		Men		Women						
	Example	*M*	*SE*	*M*	*SE*	*t*	*df*	*P*	*R^2^m*	*R^2^c*
Positive emotion terms (%)	*Brave, success*	2.13	0.0327	2.15	0.0327	−4.49	30,637	< 0.001	6.44E-05	0.453
		[2.07, 2.20]		[2.09, 2.22]						
Negative emotion terms (%)	*Bad, sick*	1.39	0.0126	1.41	0.0127	−4.73	37,196	< 0.001	1.65E-04	0.198
		[1.36, 1.41]		[1.39, 1.44]						
Body terms (%)	*Face, spine*	1.90	0.0302	1.86	0.0303	6.28	32,761	< 0.001	3.01E-04	0.400
		[1.84, 1.96]		[1.80, 1.92]						
Impersonal pronouns (%)	*It, who*	0.70	0.0136	0.71	0.0136	−2.35	28,316	.019	2.20E-05	0.281
		[0.68, 0.73]		[0.68, 0.73]						
Analytic thinking	–	92.88	0.0806	92.80	0.0808	5.94	27,472	< 0.001	9.31E-05	0.305
		[92.72, 93.04]		[92.64, 92.96]						
Cognitive processes (%)	*Affect, solve*	4.63	0.0310	4.67	0.0312	−4.38	30,241	< 0.001	5.64E-04	0.176
		[4.57, 4.69]		[4.61, 4.73]						

Note: these results include various controls reported in the main text and account for ethnicity as a fixed effect in the linear mixed model calculations. Numbers in brackets are 95% CI for the estimated marginal group means and not the difference between the group means, which are used to assess statistical significance. Full model outputs are in the online supplement. *R^2^m* refers to the marginal *R^2^*, which accounts for variance explained by the fixed effects in linear mixed model calculations (gender and ethnicity). *R^2^c* refers to the conditional *R^2^*, which accounts for variance explained by the fixed and random effects in linear mixed model calculations.

Consistent with H_2_, physicians writing about men focused more on their body (e.g. words such as *spine*and*skull*) than physicians writing about women (*P *< 0.001). For patients with related conditions, a physician’s note for a White man stated “pain poorly controlled with intermittent morphine. moaning, poor cough effort, taking shallow breaths” with one body word (*breaths*) compared to a physician’s note for a White woman from the same physician that states, “c/o severe incisional pain at rest despite morphine but lethargic after earlier doses. states she has a low pain threshold & her pain is ‘50’ on the pain scale” (zero body words). Collectively, the emotion and body words data reveal disparities in attention as modified by patient gender: physicians psychologically focus more on emotion when they attend to women (compared to men) and more on the patient’s body when they attend to men (compared to women). These differences are systematic according to the statistical evidence and consistent with qualitative examples pulled from the archive.

Consistent with H_3_, physicians used more impersonal pronouns when attending to women vs. men (*P *= 0.019). For example, a physician writing about a Black/African woman stated, “This unfortunate 44 yr old woman returns,“ which contains one impersonal pronoun (*this*) and increases the psychological distance between the physician and the patient (referring to the patient as *this* woman instead of using their name or a personal pronoun). A physician attending to a Black/African man with a related condition stated, “He was able to sleep for a few hours,” which does not contain an impersonal pronoun and instead, comparatively humanizes the patient by including a personal pronoun (*he*). This evidence suggests physicians tend to psychologically distance themselves more from women than men as revealed by pronoun patterns. Further interrogation of the impersonal pronouns and gender effect is reported in the supplementary materials.

Finally, physicians thought in more analytical and structured terms (e.g. using more articles and prepositions relative to pronouns and storytelling words) when attending to men compared to women (*P *< 0.001). A physician attending to a Black man wrote, “Sinus rhythm with atrial premature beats. Since the previous tracing probably no significant change other than the atrial premature beats,” which scored high on the analytic thinking index (98.58). A physician attending to a Black woman patient with the same diagnosis stated, “Irregular sinus tachycardia Septal + lateral ST-T changes cannot exclude myocardial ischemia,” which scored lower on the analytic thinking index (62.04). In these examples, the physician who communicated with high rates of analytic thinking used more articles and prepositions relative to storytelling words (e.g. negations) when attending to the man vs. woman.

Physicians also used fewer cognitive processing terms (e.g. indicators related to “working through” an issue or diagnosis) when attending to men compared to women (*P *< 0.001). For patients with the same diagnosis, a physician attending to a Black/African woman stated, “Baseline artifact Regular rhythm—mechanism uncertain—probably sinus rhythm although baseline artifact makes assessment difficult Low limb lead QRS voltages Otherwise baseline artifact makes assessment difficult” (seven cognitive processing words: *uncertain, probably, although, makes, lead, otherwise*, and *makes*). A physician attending to a Black/African man stated “Baseline artifact makes proper interpretation difficult. Probable sinus tachycardia. Early transition with anteroseptal ST segment depression—consider ischemia” (four cognitive processing words: *makes, interpretation, probable*, and*consider*). Less cognitive effort is required to work through diagnoses for men compared to women, a signal that physicians may be more familiar with or comfortable attending to men vs. women. Therefore, this cumulative evidence suggests gender disparities are revealed in the language of physicians who attend to different patients.

### Linguistic indicators of ethnicity bias

The data revealed systematic evidence of ethnicity bias as well, after controlling for patient gender, their diagnosis, and multiple observations by the same patient, hospital stay, and physician. For each language dimension, the main effect of ethnicity was significant *F*s > 3.83, *p*s < 0.004. Estimated marginal means and confidence intervals are reported in Table 3 and Table S8 displays *Bonferroni*-corrected mean differences out of space considerations.

**Table 3.** Estimated marginal means by ethnicity across language dimensions.

Language dimension	Ethnicity	*M*	*SE*	*df*	95% CI
Positive emotion terms (%)	Asian	2.11	0.034	2,415	[2.05, 2.18]
	Black/African	2.12	0.033	2,080	[2.05, 2.18]
	Hispanic or Latino	2.15	0.034	2,318	[2.09, 2.22]
	Other	2.17	0.033	2,015	[2.11, 2.24]
	White	2.15	0.033	1,937	[2.09, 2.22]
Negative emotion terms (%)	Asian	1.36	0.018	11,907	[1.33, 1.40]
	Black/African	1.39	0.014	4,900	[1.36, 1.41]
	Hispanic or Latino	1.42	0.017	9,903	[1.39, 1.46]
	Other	1.40	0.013	3,782	[1.38, 1.43]
	White	1.42	0.012	2,477	[1.40, 1.45]
Body terms (%)	Asian	1.97	0.035	3,770	[1.90, 2.03]
	Black/African	1.79	0.031	2,595	[1.73, 1.85]
	Hispanic or Latino	1.91	0.034	3,465	[1.84, 1.97]
	Other	1.89	0.031	2,369	[1.83, 1.95]
	White	1.87	0.030	2,094	[1.81, 1.93]
Impersonal pronouns (%)	Asian	0.699	0.015	2,709	[0.67, 0.73]
	Black/African	0.706	0.014	2,068	[0.68, 0.73]
	Hispanic or Latino	0.699	0.015	2,517	[0.67, 0.73]
	Other	0.715	0.014	1,964	[0.69, 0.74]
	White	0.702	0.014	1,814	[0.68, 0.73]
Analytic thinking	Asian	92.85	0.089	2,835	[92.68, 93.02]
	Black/African	92.77	0.083	2,147	[92.60, 92.93]
	Hispanic or Latino	92.82	0.087	2,631	[92.65, 92.99]
	Other	92.85	0.081	2,029	[92.69, 93.01]
	White	92.90	0.080	1,872	[92.75, 93.06]
Cognitive processes (%)	Asian	4.75	0.041	7,261	[4.67, 4.83]
	Black/African	4.76	0.034	3,495	[4.69, 4.82]
	Hispanic or Latino	4.65	0.039	6,151	[4.57, 4.72]
	Other	4.46	0.032	2,880	[4.39, 4.52]
	White	4.63	0.030	2,152	[4.57, 4.69]

Note: these results include various controls reported in the main text and account for gender as a fixed effect in the linear mixed model calculations. Full model outputs are in the online supplement and all *Bonferroni*-corrected mean differences are represented in Table S8. Numbers in brackets are 95% CI for the estimated marginal group means and not the difference between the group means, which are used to assess statistical significance.

Physicians attending to Black/African and Asian patients used fewer positive and negative emotion terms than physicians attending to White patients (*p*s < 0.001). Example texts from patients with the same diagnosis and gender, but different ethnicity, demonstrate clear differences in how patients are discussed and the disparities that exist. A physician for a Black/African man wrote, “Infant observed during cares. Developmental care plan posted at the bedside. Please refer to for details on infant strengths, stress signals, and ways to optimize infant comfort. OT to follow,” which contains one negative emotion term (*stress*) and abstractly describes a patient’s care (e.g. a care plan was “posted,” instead of detailing the care plan). A physician for a White man wrote “pt occationally irritable tonight with increased gas. tylenol given for circumcision discomfort as witnessed by facial grimacing and increased heart rate,” which contains three negative emotion terms (*irritable, discomfort*, and *grimacing*) and more completely describes physical conditions and experiences of the patient. Therefore, physicians focus less on the pain and negative experiences of Black/African patients compared to White patients as revealed by language. These effects are generally consistent with nonlanguage research findings as well, where physicians believe Black patients can generally feel less pain than White patients (57, 58). It is also important to note that physicians used the lowest rate of body terms when attending to Black/African patients compared to patients of other ethnicities (*p*s < 0.001; see Table 3). Collectively, this evidence suggests that through their language and caregiver notes, physicians focus less on the emotions and physical diagnoses of Black/African patients compared to most patients of other ethnicities.

Physicians attending to Black/African patients also thought in less structured and analytical terms compared to physicians attending to White patients (see Table 3 and Table S8). Further, physicians used fewer cognitive processing terms for White patients (“Sinus tachycardia Possible left atrial abnormality Since last ECG, no significant change;“ four cognitive processing terms: *possible, abnormality, since*, and *change*) compared to Asian patients (“Atrial fibrillation. Modest ST-T wave changes are non-specific. Since the previous tracing of [**deidentified**] ventricular rate is slower. Otherwise, probably no significant change;“ six cognitive processing terms: *changes, specific, since, otherwise, probably*, and *change*) and Black/African patients (“Normal sinus rhythm. Probable lead reversal between lead V1–V2. Occasional ventricular premature beat. Compared to tracing #1, no change other than lead reversal;” eight cognitive processing terms: *probable, lead, lead*,*occasional, change, other, than*, and*lead*). Note, all patients in the prior examples were women and had the same diagnosis. The cognitive processing evidence suggests physicians may need to expend less cognitive effort to work through diagnoses and organize their thoughts for White patients vs. non-White patients. Word patterns reveal cognitive correlates of treating patients from different ethnic backgrounds.

Finally, as the evidence in Table S8 suggests, linguistic differences across ethnicities for impersonal pronouns were largely nonsignificant. Bias and language-based disparities mainly occur through other linguistic pathways in medicine, including emotion and cognition.

### Exploratory content patterns

**Fig. 1.**
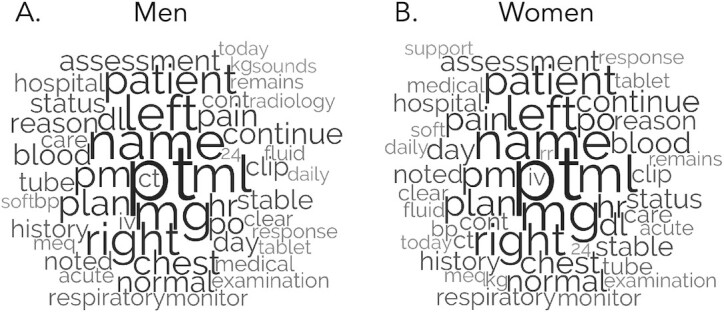
Note. Font size reflects relative raw prevalence of words within groups.

To further understand the linguistic disparities that exist across major gender and ethnicity groups, an exploratory content analysis was performed. Word clouds in Fig. 1 indicate the 50 most frequent content words across genders. Supported by the evidence also presented in Table S9, the data suggest content differences across men and women were relatively minor. Indeed, 48/50 of the most frequent content words appeared in both men and women lists (e.g. only four words were not cross-listed: *radiology, sounds, rr*, and *support*). Physicians focus on patients of different genders with generally stable content, further emphasizing the importance of style patterns reported in this work as well.

**Fig. 2.**
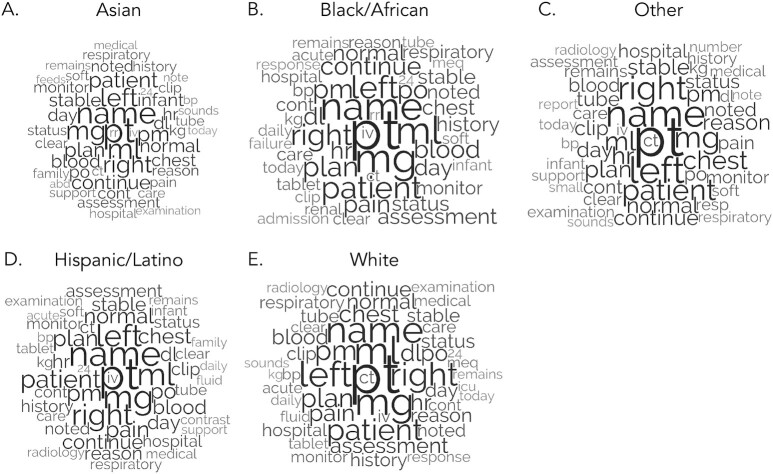
Note. Font size reflects relative raw prevalence of words within groups.

The content in caregiver notes was more distinct and variable across ethnicities, however (see Fig. 2 and Table S10, which provide complementary descriptive details on content patterns). In one example, the word *pain—*a negative emotion term—appeared in 22.66% of caregiver notes for White patients, 19.77% of caregiver notes for Black/African patients, and 14.73% of caregiver notes for Asian patients. Given the scale of these data, such percentage differences reflect a nontrivial number of caregiver notes that mention the term *pain* across ethnicities. Further, diagnoses of White, Black/African, and Hispanic or Latino patients were often described as *acute*, but this term was not in the top 50 content words for Asian patients and those in the “Other” category. In fact, *acute* appeared in 19.23% of caregiver notes for White patients, 18.13% of caregiver notes for Black/African patients, 15.99% of caregiver notes for Hispanic or Latino patients, and 14.89% of caregiver notes for Asian patients. The term *acute* is often used to describe the sudden and severe onset of a condition (59), which may represent a disparity in how physicians think about health conditions and their association with time for patients of different ethnicities (e.g. how long might take for a patient to recognize they need medical attention). Together, this evidence suggests physicians focus on different content when attending to the medical and health experiences for patients of varying ethnicities in critical care. Please see the supplementary materials for more content differences across gender and ethnicities.

### Gender × ethnicity bias interaction

To investigate the joint impact of patient gender and ethnicity on physician word patterns in caregiver notes, mixed effect interactions with the prior controls were conducted. The only interaction effect models to reach statistical significance were for cognitive processes, *F*(4,30,195) = 2.92, *P *= 0.019, and positive emotion terms, *F*(4,30,726) = 4.17, *P *= 0.002. Key findings are represented below and estimated marginal means (plus mean differences) across groups are in the supplementary materials out of space considerations (see Tables S11–S17).

Physicians wrote with the greatest rate of cognitive processing terms for Black/African women (see Table S11). An example of a physician working through a diagnosis for a Black/African woman includes, “Sinus rhythm. Since the previous tracing of [**deidentified**] inferolateral ST-T wave abnormalities may be less. Otherwise, no change.,” which includes five cognitive processing terms (*since, abnormalities, may, otherwise*, and*change*). This example demonstrates a degree of uncertainty in the patient’s progress, where the physician needs to work through and put in additional cognitive effort to attend to their care. Comparatively, a physician describing a White woman with the same condition stated, “Ventricular paced rhythm Since previous tracing, atrial pacer spikes are not as apparent,” with three cognitive processing terms (*since, not*, and *apparent*) and is more certain in its evaluation of the patient. *Bonferroni*-corrected multiple comparisons revealed significant differences between Black/African women and all other groups (*p*s < 0.015), except for Asian men. Together, physicians demonstrate the greatest need to work through diagnoses for Black/African women, whereas patients of other genders and ethnicities received less questioning and required less cognitive effort from caregivers.

Finally, physicians of Black/African women focused on less positive affect than physicians of White women (*P *< 0.001). Black/African women, on average, were described with the lowest rate of positive affect compared to other patients.

## Discussion

Bias pervades many aspects of social life (60), and the current work demonstrates systematic gender and ethnicity disparities in medicine through language. The evidence suggests physicians focus more on impersonality and emotion when attending to women compared to men, physicians attend less to the negative experiences of Black/African and Asian patients than White patients, and physicians expend more cognitive effort to work through issues for Black/African women vs. other groups of people. These observations build on nonlinguistic findings that indicate how inequalities are widespread in medicine (5, 57, 58), and this study is among the first to demonstrate the effects are robust in language patterns among 1.8 million caregiver notes and over 500 million words from such reports.

This evidence is important because it establishes a link between communication patterns and bias that is often unobserved or underexamined in medicine. Bias in medicine has been predominantly revealed through procedural differences among ethnic groups (4), how patients of different ethnicities perceive their medical treatment (47), and structures that are barriers-to-entry for women and ethnic minorities (61). The current work revealed that the language found in everyday caregiver notes reflects disparities and indications of bias—new pathways that can complement other approaches to signal physicians who treat patients inequitably (62). Caregiver notes, based on their private nature, are akin to medical diaries for physicians as they attend to patients, logging the thoughts, feelings, and diagnoses of medical professionals. Caregivers have the herculean task of tending to those in need, though the current evidence suggests bias and language-based disparities are a part of this system. Words might therefore be used to alert physicians when they are in settings that can facilitate bias. The results in the current work are critical because language is inherently linked to how people think and feel about others; believing that certain groups are “just more emotional” (women vs. men) or certain groups are unable to feel (Black/African and Asian patients vs. White patients), may make physicians underestimate the pain of some patients and underserve their care. Ultimately, this work does not intend to accuse or cast blame on specific people, but to unearth disparities and biases with the hope of mitigating them in a pursuit of equity.

Against this backdrop, it is also important to position the observed linguistic disparities within prior stereotyping and bias research. First, in the current sample via content words, physicians focused less on *acute pain* of Asian patients compared to other ethnicities. Prior work suggests stereotypes of Asian people often emphasize their competence but deemphasize their warmth (44, 63), which might also be present in medicine when physicians attend less to their pain and the acuity of their symptoms (e.g. the distress of Asian patients was under-responded to) (43). Therefore, this work complements and extends traditional stereotyping and bias research by offering pathways to indicate how established disparities are revealed in the language of medical records. Second, in the United States, prior work suggests Asian people are often feminized and Black people tend to be masculinized (64, 65). This pattern of stereotyping makes Black women and Asian men the nonprototypical members of their racial groups (66, 67), which, therefore, may require physicians to exert more cognitive effort to work through their diagnoses compared to prototypical members of their racial groups. The interaction effect analyses support such contentions, where rates of cognitive processing terms were highest for Black women and Asian men. Altogether, consistent with stereotype content model research, stereotypes and biases are systematic in intergroup settings; the current work expands on this foundation to observe how these patterns are revealed linguistically and at scale in medicine.

A critical reader might question if the reported effects are indeed a reflection of bias, or perhaps other psychological, institutional, or communication processes. There are several reasons why the effects are likely bias instead of other alternatives. First, the evidence in this paper is consistent with patterns of gender and ethnicity bias observed in other work. Language patterns, therefore, are another way that bias is represented at scale and naturally, from actual patient–physician records. Second, the effect sizes are small, which is consistent with how gender and ethnicity bias is generally communicated or revealed in the wild. Overt misogyny and discrimination are relatively rare compared to subtle or everyday forms of bias that accumulate over time (68, 69). The differences between genders and ethnicities are indeed disparities, where specific psychological attention is paid to some groups and not others, as revealed through a linguistic signature of physician data.

Relatedly, it is also important to position the cognitive processing results from this study, which suggest physicians displayed more evidence of “working through” medical cases of Black/African women compared to other patients. It could be argued that this additional cognitive effort is a sign of physicians trying to understand Black/African women better (e.g. putting in more cognitive effort to give better care). However, considering this evidence with the other linguistic findings (e.g. less emotional focus) reveals a less inclusive picture. Physicians needing to expend more cognitive effort for Black/African women compared to other patient groups is also consistent with established evidence suggesting Black/African patients, in general, are unheard and misunderstood medically compared to others (47, 70). Here, expending more cognitive effort likely reflects unfamiliarity and uncertainty instead of advocacy, especially as Black women often have worse health outcomes than other groups (71).

Taken together, bias in medicine and its associated linguistic disparities are systematic, nuanced, and contingent on a particular group in question. For example, an increase in one linguistic feature (e.g. negative emotion terms) may be a signal of bias toward some groups (e.g. women vs. men) while a decrease in the same linguistic feature may be a signal of bias toward other groups (e.g. Black/African and Asian patients vs. White patients). Linguistic bias, therefore, depends on the group being examined and requires a historical and contextual understanding of how groups have been treated in medicine to reveal patterns of mistreatment. The evidence in this paper was often consistent with other accounts revealing medical stereotypes by gender (e.g. women are “just more emotional” than men) and ethnicity (e.g. Black patients are less capable of feeling pain compared to White patients), though the current work demonstrates new links to bias at the language level. Medicine’s persistent struggle to treat women and non-White individuals equitably suggests new ways to understand and mitigate disparities are worthwhile, pressing, and needed for system-level change.

The emotion and body words evidence also reveal how bias can manifest in compensatory ways in medicine. Physicians’ attention is limited when focusing on patients of different genders and ethnicities. By focusing on one aspect of a patient (e.g. their emotions), they are often unable to adequately focus other important aspects of their care for some groups (e.g. their body and physical diagnoses). For example, as positive and negative emotion terms increased in physicians notes for women (compared to men), body words decreased as well. As positive and negative emotion terms decreased in physician notes for Asian patients (compared to White patients), body words increased. Therefore, by focusing more (or less) on one aspect of a patient, physicians may psychologically compensate and have less (or more) ability to attend to other aspects of their health. This is a novel theoretical insight into bias and linguistic disparities, which deserves further interrogation into why it occurs for some groups and not others.

### Limitations and future directions

In light of this evidence, there are limitations of this work worth noting. First, these data are correlational, and future work should use experimental procedures to identify how physicians might write caregiver reports based on patient profiles. Psychological mechanisms are also critical to examine with additional research. Second, the effect sizes in this paper are small, and identifying these patterns benefitted from the large number of cases available in the dataset. To contextualize the effect sizes, however, the word counting approach applied to caregiver notes was simple and most effect sizes in this paper are consistent with psychology of language research (72). LIWC’s simple word counting system offers a baseline level of bias identification and more sophisticated procedures will likely identify gender and ethnicity bias in new and more predictive ways. Further, these data were only collected from one hospital, and it is unclear if physician demographics at Beth Israel Deaconess Medical Center (e.g. those who wrote the caregiver reports) are typical of most hospitals in the Unite States. Future research should collect caregiver reports from more hospitals to identify how such language patterns of bias represent system-level or regional tendencies. Due to data availability limitations, physician demographics could not be accounted for and, therefore, it is unclear who perpetuated bias toward women and people of color. This nuance should be examined in future research if such data are obtainable. Finally, while the reported effects demonstrate clear connections to bias and language-based disparities, there are other possible explanations that deserve treatment as well. The patterns of emotion, for example, may result from a form of mimicry or matching where physicians are reflecting a patient’s communication style back to them. This is still bias, however, as physicians are often told to be objective, detached, and unemotional in their assessment of patients (73, 74), but this aim is not universally achieved. Additional work should examine related social and psychological dynamics to understand how bias manifests in medical settings and medicine as an institution.

Future work would also benefit from exploring how bias, over the course of a patient’s medical progress, is reflected over time and across groups. Physicians may focus on different aspects of a patient’s health experience depending on their gender and ethnicity during a hospital admittance, and this progression may have implications for the quality and urgency of their care. Additionally, it may be important to evaluate general baseline rates of emotion (and other language variables) in medical settings. Such baselines can serve as “ground truth” to consider how the current results compare to some general assessment of how physicians communicate about groups of people. Nonetheless, these data indicate disparities in how physicians attend to patients based on their gender and ethnicity. While the results indicate disparities in how physicians focus on their patients of different subgroups, future work should identify how such disparities lead to different treatments and health outcomes as well. The link between gender, ethnicity, language, and bias might be more consequential by observing that disparities lead to inequitable care or treatment, plus worse medical outcomes.

## Supplementary Material

pgac157_Supplemental_File

## Data Availability

Per the terms of the data acquisition agreement (https://physionet.org/content/mimiciii/view-license/1.4/), the data cannot be shared publicly. For access to the MIMIC-III database, please visit the following site, https://physionet.org/content/mimiciii/1.4/, or contact one of the database maintainers, Dr Tom Pollard (tpollard@mit.edu). Sample statistical code for the linear mixed models is available on the Open Science Framework: https://osf.io/eu3tr/.

## Footnotes

a Some studies recommend removing especially low word counts to prevent denominator effects, or the idea that some language categories may be overstated when there is a small number of words in a piece of text. Excluding texts ≤ 20 words (*N *= 1,774,170) produced substantively equivalent results and comparable effect sizes in this paper.

b The unit-weighted formula for calculating the analytic thinking index is: analytic thinking = [articles + prepositions—pronouns—auxiliary verbs—adverb—conjunctions—negations] from LIWC scores (55). Algorithmically, the analytic thinking index is then created by normalizing the unit-weighted result. Such normalized scores are “percentiles based on standardized scores from large comparison corpora” (56) and range from 0, or low analytic thinking, to 100, or high analytic thinking.

c Some physician IDs were blank and to prevent an abundance of missing values, were collapsed into a single level (a single physician ID).

d According to the database creators, gender refers to the genotypical sex of the patient. Therefore, gender is a biological construct in this study.

e In the raw data, ethnicity was more granularly reported. However, ethnicities were collapsed into dominant categories for simplicity of the statistical models. The “other” category also included patients who did not disclose their ethnicity or whose ethnicity could not be determined.

f Prior work suggests bias against Black people persists even after controlling for perpetrator ethnicity as well (20). Therefore, not having physician gender or ethnicity is noteworthy, but not fatal for the analyses.

g In some cases, excerpts were not full caregiver notes due to their length. However, no words were removed in the process of identifying such exemplars. Excerpts are used to highlight how the language dimensions operated in actual caregiver notes across groups.

## Author’s note

The author acknowledges and appreciates that gender and sex are distinct concepts. In this study, gender refers to the genotypical sex of the patient, and the term gender was kept because it was used by the creators of the MIMIC-III database.

## References

[1] Hamberg K . 2008. Gender bias in medicine. Womens Health. 4:237–243. 10.2217/17455057.4.3.237 19072473

[2] Samulowitz A , Gremyr I, Eriksson E, Hensing G. 2018. “Brave men” and “emotional women”: a theory-guided literature review on gender bias in health care and gendered norms towards patients with chronic pain. Pain Res Manag. 2018:1–14. 10.1155/2018/6358624 PMC5845507 29682130

[3] Onyeador IN et al. 2019. The value of interracial contact for reducing anti-black bias among non-black physicians: a cognitive habits and growth evaluation (CHANGE) study report. Psychol Sci. 31:18–30. 31743078 10.1177/0956797619879139 PMC6966250

[4] Schulman KA et al. 1999. The effect of race and sex on physicians’ recommendations for cardiac catheterization. N Engl J Med. 340:618–626. 10029647 10.1056/NEJM199902253400806

[5] Mossey JM . 2011. Defining racial and ethnic disparities in pain management. Clin Orthop. 469:1859–1870. 21249483 10.1007/s11999-011-1770-9 PMC3111792

[6] Santry HP , Wren SM. 2012. The role of unconscious bias in surgical safety and outcomes. Surg Clin. 92:137–151. 10.1016/j.suc.2011.11.006 PMC3417145 22269267

[7] Williams DR , Wyatt R. 2015. Racial bias in health care and health: challenges and opportunities. JAMA. 314:555–556. 26262792 10.1001/jama.2015.9260

[8] Beach MC et al. 2021. Testimonial injustice: linguistic bias in the medical records of black patients and women. J Gen Intern Med. 36:1708–1714. 33754318 10.1007/s11606-021-06682-z PMC8175470

[9] Hagiwara N , Slatcher RB, Eggly S, Penner LA. 2016. Physician racial bias and word use during racially discordant medical interactions. Health Commun. 32:401–408. 27309596 10.1080/10410236.2016.1138389 PMC5161737

[10] Pennebaker JW . 2011. The secret life of pronouns: what our words say about us. London: Bloomsbury Press.

[11] Pennebaker JW . 1997. Opening up: the healing power of expressing emotions. New York (NY): The Guilford Press.

[12] Boyd RL , Schwartz HA. 2021. Natural language analysis and the psychology of verbal behavior: the past, present, and future states of the field. J Lang Soc Psychol. 40:21–41. 34413563 10.1177/0261927x20967028 PMC8373026

[13] Tausczik YR , Pennebaker JW. 2010. The psychological meaning of words: LIWC and computerized text analysis methods. J Lang Soc Psychol. 29:24–54.

[14] Boyd RL , Pennebaker JW. 2015. A way with words: using language for psychological science in the modern era. In: Dimofte C, Haugtvedt C, Yalch R, editors. Consumer psychology in a social media world. Oxfordshire: Routledge. p. 222–236.

[15] Kennedy B , Ashokkumar A, Boyd RL, Dehgani M. 2022. Text analysis for psychology: methods, principles, and practices. In: Dehghani M, Boyd RL, editors. Handbook of language analysis in psychology. Guilford Press.

[16] Kacewicz E , Pennebaker JW, Davis M, Jeon M, Graesser AC. 2014. Pronoun use reflects standings in social hierarchies. J Lang Soc Psychol. 33:125–143.

[17] Ireland ME , Mehl M. 2014. Natural language use as a marker of personality. In: Holtgraves TM, editor. The oxford handbook of language and social psychology. Oxford: Oxford University Press.

[18] Markowitz DM , Slovic P. 2020. Social, psychological, and demographic characteristics of dehumanization toward immigrants. Proc Natl Acad Sci. 117:9260–9269. 32300012 10.1073/pnas.1921790117 PMC7196895

[19] Mendelsohn J , Tsvetkov Y, Jurafsky D. 2020. A framework for the computational linguistic analysis of dehumanization. Front Artif Intell. 0:55. 10.3389/frai.2020.00055 PMC7861242 33733172

[20] Voigt R et al. 2017. Language from police body camera footage shows racial disparities in officer respect. Proc Natl Acad Sci. 114:6521–6526. 28584085 10.1073/pnas.1702413114 PMC5488942

[21] Charlesworth TES , Yang V, Mann TC, Kurdi B, Banaji MR. 2021. Gender stereotypes in natural language: word embeddings show robust consistency across child and adult language corpora of more than 65 million words. Psychol Sci. 32:218–240. 33400629 10.1177/0956797620963619

[22] Caliskan A , Bryson JJ, Narayanan A. 2017. Semantics derived automatically from language corpora contain human-like biases. Science. 356:183–186. 28408601 10.1126/science.aal4230

[23] Bartley EJ , Fillingim RB. 2013. Sex differences in pain: a brief review of clinical and experimental findings. Br J Anaesth. 111:52–58. 23794645 10.1093/bja/aet127 PMC3690315

[24] Zhang L , Losin EAR, Ashar YK, Koban L, Wager TD. 2021. Gender biases in estimation of others’ pain. J Pain. 22:1048–1059. 33684539 10.1016/j.jpain.2021.03.001 PMC8827218

[25] Wesolowicz DM , Clark JF, Boissoneault J, Robinson ME. 2018. The roles of gender and profession on gender role expectations of pain in health care professionals. J Pain Res. 11:1121–1128. 29942147 10.2147/JPR.S162123 PMC6007196

[26] Schäfer G , Prkachin KM, Kaseweter KA, de C Williams AC. 2016. Health care providers’ judgments in chronic pain: the influence of gender and trustworthiness. Pain. 157:1618–1625. 26934512 10.1097/j.pain.0000000000000536

[27] Wallace B et al. 2021. Towards health equity for people experiencing chronic pain and social marginalization. Int J Equity Health. 20:53. 33531018 10.1186/s12939-021-01394-6 PMC7852178

[28] Chen EH et al. 2008. Gender disparity in analgesic treatment of emergency department patients with acute abdominal pain. Acad Emerg Med. 15:414–418. 18439195 10.1111/j.1553-2712.2008.00100.x

[29] Fu L , Danescu-Niculescu-Mizil C, Lee L, 2016. Tie-breaker: using language models to quantify gender bias in sports journalism. Proceedings of the IJCAI Workshop on NLP Meets Journalism. March 16, 2022. New York.

[30] Foss C . 2002. Gender bias in nursing care? Gender-related differences in patient satisfaction with the quality of nursing care. Scand J Caring Sci. 16:19–26. 11985745 10.1046/j.1471-6712.2002.00045.x

[31] Foss C , Sundby J. 2003. The construction of the gendered patient: hospital staff's attitudes to female and male patients. Patient Educ Couns. 49:45–52. 12527152 10.1016/s0738-3991(02)00039-3

[32] Wilson J . 1990. Politically speaking: the pragmatic analysis of political language. Oxford: Basil Blackwell.

[33] Markowitz DM , Slovic P. 2020. Communicating imperatives requires psychological closeness but creates psychological distance. J Lang Soc Psychol. 39:598–625.

[34] Weiner M , Mehrabian A, 1968. Language within language: immediacy, a channel in verbal communication. London: Ardent Media.

[35] Zheni T . 2020. Person deixis as biased political pronouns in George W. Bush's speeches on Iraqi War II. Int J Lang Lit Stud. 2:155–171.

[36] Kahneman D . 2011. Thinking, fast and slow. New York (NY): Farrar, Straus and Giroux.

[37] Pennebaker JW , Chung CK, Frazee J, Lavergne GM, Beaver DI. 2014. When small words foretell academic success: the case of college admissions essays. PLoS ONE. 9:e115844. 25551217 10.1371/journal.pone.0115844 PMC4281205

[38] Markowitz DM . 2022. Psychological trauma and emotional upheaval as revealed in academic writing: the case of COVID-19. Cogn Emot. 36:9–22. 34965201 10.1080/02699931.2021.2022602

[39] Seraj S , Blackburn KG, Pennebaker JW. 2021. Language left behind on social media exposes the emotional and cognitive costs of a romantic breakup. Proc Natl Acad Sci. 118:e2017154118. 33526594 10.1073/pnas.2017154118 PMC7896325

[40] Boyd RL , Blackburn KG, Pennebaker JW. 2020. The narrative arc: revealing core narrative structures through text analysis. Sci Adv. 6:eaba2196. 32821822 10.1126/sciadv.aba2196 PMC7413736

[41] Hsu KJ , Babeva KN, Feng MC, Hummer JF, Davison GC. 2014. Experimentally induced distraction impacts cognitive but not emotional processes in think-aloud cognitive assessment. Front Psychol. 5:474. 24904488 10.3389/fpsyg.2014.00474 PMC4033004

[42] Ashokkumar A , Pennebaker JW. 2021. Social media conversations reveal large psychological shifts caused by COVID-19’s onset across U.S. cities. Sci Adv. 7:7843–7865. 10.1126/sciadv.abg7843 PMC8457655 34550738

[43] Zou LX , Cheryan S. 2017. Two axes of subordination: a new model of racial position. J Pers Soc Psychol. 112:696–717. 28240941 10.1037/pspa0000080

[44] Fiske ST , Cuddy AJC, Glick P, Xu J. 2002. A model of (often mixed) stereotype content: competence and warmth respectively follow from perceived status and competition. J Pers Soc Psychol. 82:878–902. 12051578

[45] Fiske ST . 2012. Warmth and competence: stereotype content issues for clinicians and researchers. Can Psychol Can. 53:14–20. 10.1037/a0026054 PMC3801417 24155504

[46] Fiske ST . 2018. Stereotype content: warmth and competence endure. Curr Dir Psychol Sci. 27:67–73. 29755213 10.1177/0963721417738825 PMC5945217

[47] Beach MC , Branyon E, Saha S. 2017. Diverse patient perspectives on respect in healthcare: a qualitative study. Patient Educ Couns. 100:2076–2080. 28602565 10.1016/j.pec.2017.05.010 PMC6400635

[48] Wolsiefer KJ et al. 2021. Investigating the relationship between resident physician implicit bias and language use during a clinical encounter with hispanic patients. Health Commun. 1–9.. DOI: 10.1080/10410236.2021.1936756. PMC9524003 34130567

[49] Johnson AEW et al. 2016. MIMIC-III, a freely accessible critical care database. Sci Data. 3:1–9. 10.1038/sdata.2016.35 PMC4878278 27219127

[50] Johnson AEW , Pollard TJ, Mark RG. 2016. MIMIC-III clinical database (version 1.4). Sci Data. 3:160035. PhysioNet. DOI: 10.13026/C2XW26. 27219127 PMC4878278

[51] Goldberger AL et al. 2000. PhysioBank, PhysioToolkit, and PhysioNet. Circulation. 101:E215–20. 10851218 10.1161/01.cir.101.23.e215

[52] Pennebaker JW , Booth RJ, Boyd RL, Francis ME, 2015. Linguistic inquiry and word count. LIWC2015. Mahwah (NJ): Lawrence Erlbaum Associates, Incorporated.

[53] Boyd RL . 2017. Psychological text analysis in the digital humanities. In: Hai-Jew S, editor. Data analytics in digital humanities. New York (NY): Springer International Publishing. p. 161–189.

[54] Kennedy B , Ashokkumar A, Boyd RL, Dehghani M, 2022. Text analysis for psychology: methods, principles, and practices. In: Dehghani M, Boyd RL, editors. Handbook of language analysis in psychology. New York (NY): Guilford Press.

[55] Jordan KN , Sterling J, Pennebaker JW, Boyd RL. 2019. Examining long-term trends in politics and culture through language of political leaders and cultural institutions. Proc Natl Acad Sci USA. 116:3476–3481. 30808741 10.1073/pnas.1811987116 PMC6397582

[56] Boyd RL , Ashokkumar A, Seraj S, Pennebaker JW, 2022. The development and psychometric properties of LIWC-22. Austin (TX): University of Texas at Austin.

[57] Hoffman KM , Trawalter S, Axt JR, Oliver MN. 2016. Racial bias in pain assessment and treatment recommendations, and false beliefs about biological differences between blacks and whites. Proc Natl Acad Sci. 113:4296–4301. 27044069 10.1073/pnas.1516047113 PMC4843483

[58] Cintron A , Morrison RS. 2006. Pain and ethnicity in the United States: a systematic review. J Palliat Med. 9:1454–1473. 17187552 10.1089/jpm.2006.9.1454

[59] National Library of Medicine . 2022. Acute vs. chronic conditions: MedlinePlus medical encyclopedia image. Bethesda (MD). March 19, 2022.

[60] Eberhardt JL . 2019. Biased: uncovering the hidden prejudice that shapes what we see, think, and do. New York (NY): Penguin Books.

[61] Hadinger MA . 2016. Underrepresented minorities in medical school admissions: a qualitative study. Teach Learn Med. 29:31–41. 27824260 10.1080/10401334.2016.1220861

[62] Greenwald AG , McGhee DE, Schwartz JLK. 1998. Measuring individual differences in implicit cognition: the implicit association test. J Pers Soc Psychol. 74:1464–1480. 9654756 10.1037//0022-3514.74.6.1464

[63] Cuddy AJC , Fiske ST, Glick P. 2007. The BIAS map: behaviors from intergroup affect and stereotypes. J Pers Soc Psychol. 92:631–648. 17469949 10.1037/0022-3514.92.4.631

[64] Johnson KL , Freeman JB, Pauker K. 2012. Race is gendered: how covarying phenotypes and stereotypes bias sex categorization. J Pers Soc Psychol. 102:116–131. 21875229 10.1037/a0025335

[65] Goff PA , Thomas MA, Jackson MC. 2008. “Ain't I a woman?”: towards an intersectional approach to person perception and group-based harms. Sex Roles. 59:392–403.

[66] Purdie-Vaughns V , Eibach RP. 2008. Intersectional invisibility: the distinctive advantages and disadvantages of multiple subordinate-group identities. Sex Roles. 59:377–391.

[67] Schug J , Alt NP, Lu PS, Gosin M, Fay JL. 2017. Gendered race in mass media: invisibility of Asian men and Black women in popular magazines. Psychol Pop Media Cult. 6:222–236.

[68] Eschmann R . 2020. Unmasking racism: students of color and expressions of racism in online spaces. Soc Probl. 67:418–436.

[69] Sue DW . 2010. Microaggressions in everyday life: race, gender, and sexual orientation. Hoboken (NJ): John Wiley & Sons.

[70] Schencker L . 2020. Racism in medicine: Chicago hospitals work to fight bias, improve outcomes for Black patients. Chicago Tribune. November 3, 2021.

[71] Chinn JJ , Martin IK, Redmond N. 2021. Health equity among Black women in the United States. J Womens Health. 30:212–219. 10.1089/jwh.2020.8868 PMC8020496 33237831

[72] Kern ML et al. 2014. The online social self: an open vocabulary approach to personality. Assessment. 21:158–169. 24322010 10.1177/1073191113514104

[73] Smith AC , Kleinman S. 1989. Managing emotions in medical school: students’ contacts with the living and the dead. Soc Psychol Q. 52:56–69.

[74] Lief HI , Fox RC. 1963. Training for detached concern in medical students. In: Lief HI, Lief VF, Lief NR, editor. The psychological basis for medical practice. New York (NY): Harper & Row. p. 12–35.

