# Supplementary material for: Gender and ethnicity bias in medicine: a text analysis of 1.8 million critical care records
Source: PNAS Nexus. 2022 Aug 18;1(4):pgac157. doi: 10.1093/pnasnexus/pgac157 (PMC9802334; doi:10.1093/pnasnexus/pgac157)
Supplement: pgac157_Supplemental_File [file pgac157_supplemental_file.docx]

**
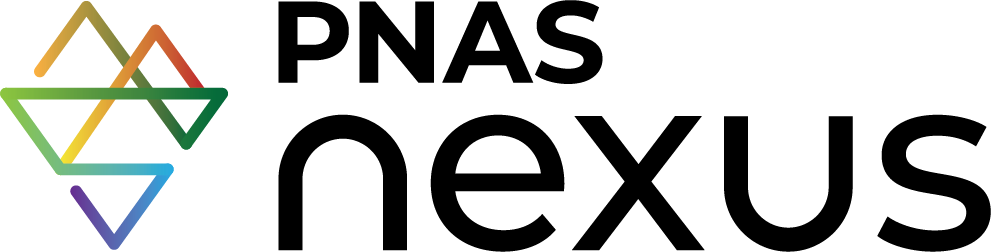
**

**Supplementary Information for**

Gender and Ethnicity Bias in Medicine: A Text Analysis of 1.8 Million Critical Care Records

David M. Markowitz

David M. Markowitz

Email: [dmark@uoregon.edu](mailto:dmark@uoregon.edu)

**This PDF file includes:**

Table of Contents

Further Interrogation of Impersonal Pronouns and Gender Finding

Tables S1 to S17

Table of Contents

[Supplementary Table S1: Correlation Matrix of Key Language Variables 3](#_Toc98830340)

[Supplementary Table S2: Positive Emotion Terms Mixed Model Results 4](#_Toc98830341)

[Supplementary Table S3: Negative Emotion Terms Mixed Model Results 5](#_Toc98830342)

[Supplementary Table S4: Body Terms Mixed Model Results 6](#_Toc98830343)

[Supplementary Table S5: Impersonal Pronouns Mixed Model Results 7](#_Toc98830344)

[Supplementary Table S6: Analytic Thinking Mixed Model Results 8](#_Toc98830345)

[Supplementary Table S7: Cognitive Processes Mixed Model Results 9](#_Toc98830346)

[Further Interrogation of Impersonal Pronouns and Gender Finding 10](#_Toc98830347)

[Supplementary Table S8: Linguistic Mean Differences Across Ethnicities 11](#_Toc98830348)

[Supplementary Table S9: Frequency of Content Words by Gender 12](#_Toc98830349)

[Supplementary Table S10: Frequency of Content Words by Ethnicity 13](#_Toc98830350)

[Supplementary Table S11: Means Across Groups for Interaction Effects 15](#_Toc98830351)

[Supplementary Table S11: Means Across Groups for Interaction Effects (Continued) 16](#_Toc98830352)

[Supplementary Table S12: Interaction Effect Mean Differences for Positive Emotion Terms 17](#_Toc98830353)

[Supplementary Table S13: Interaction Effect Mean Differences for Negative Emotion Terms 18](#_Toc98830354)

[Supplementary Table S14: Interaction Effect Mean Differences for Body Terms 19](#_Toc98830355)

[Supplementary Table S15: Interaction Effect Mean Differences for Impersonal Pronouns 20](#_Toc98830356)

[Supplementary Table S16: Interaction Effect Mean Differences for Analytic Thinking 21](#_Toc98830357)

[Supplementary Table S17: Interaction Effect Mean Differences for Cognitive Processes 22](#_Toc98830358)

# **Supplementary Table S1: Correlation Matrix of Key Language Variables**

|  | Ipron | Posemo | Negemo | Body | Analytic |
| --- | --- | --- | --- | --- | --- |
| Posemo | -0.00807^**^ |  |  |  |  |
| Negemo | -0.04911^**^ | -0.16797^**^ |  |  |  |
| Body | -0.01029^**^ | 0.07779^**^ | -0.002^**^ |  |  |
| Analytic | -0.27208^**^ | -0.04824^**^ | -0.04339^**^ | 0.02151^**^ |  |
| Cogproc | 0.08283^**^ | -0.21906^**^ | 0.15425^**^ | -0.02019^**^ | -0.15201^**^ |

*Note*. Correlations reflect Pearson’s correlations (*r*) and all relationships were statistically significant at ^**^*p* < .01. Variable names are LIWC names (ipron = impersonal pronouns, posemo = positive emotion terms, negemo = negative emotion terms, body = body terms, Analytic = analytic thinking, cogpoc = cognitive processes).

# **Supplementary Table S2: Positive Emotion Terms Mixed Model Results**

| Language dimension | Fixed effects | *B* | *SE* | *df* | *t* | *p* |
| --- | --- | --- | --- | --- | --- | --- |
| Positive emotion terms (%) | Intercept | 2.16E+00 | 3.26E-02 | 1.96E+03 | 66.306 | < .001 |
|  | Gender: Men | -1.75E-02 | 3.89E-03 | 3.06E+04 | -4.489 | < .001 |
|  | Ethnicity: Other | 1.84E-02 | 5.64E-03 | 4.04E+04 | 3.261 | .001 |
|  | Ethnicity: Asian | -4.05E-02 | 1.15E-02 | 3.41E+04 | -3.512 | < .001 |
|  | Ethnicity: Black/African | -3.62E-02 | 6.95E-03 | 2.28E+04 | -5.212 | < .001 |
|  | Ethnicity: Hispanic or Latino | -1.09E-03 | 1.05E-02 | 2.93E+04 | -0.104 | .917 |
|  | Random effects | *n* | σ^2^ | *SD* |  |  |
|  | Hospitalization ID | 58,361 | 0.035103 | 0.18736 |  |  |
|  | Subject ID | 46,139 | 0.027896 | 0.16702 |  |  |
|  | Diagnosis | 15,589 | 0.002748 | 0.05243 |  |  |
|  | Caregiver ID | 1,913 | 1.917213 | 1.38463 |  |  |
|  | *R*^2^m | 6.438845e-05 |  |  |  |  |
|  | *R*^2^c | 0.4530003 |  |  |  |  |

*Note*. The reference group for the gender fixed effect is “Women” and the reference group for the ethnicity fixed effect is “White.” *R^2^m* refers to the marginal *R^2^*, which accounts for variance explained by the fixed effects in linear mixed model calculations (gender and ethnicity). *R^2^c* refers to the conditional *R^2^*, which accounts for variance explained by the fixed and random effects in linear mixed model calculations.

These results suggest physicians use more positive emotion terms when attending to women compared to men after controlling for the patient’s ethnicity (and the random effects as well). Physicians attending to Black/African and Asian patients focus on fewer positive emotion terms compared to White patients, after controlling for patient’s gender (and the random effects as well). Please refer to Supplementary Table S8 for *Bonferroni*-corrected multiple comparisons across ethnicity categories.

# **Supplementary Table S3: Negative Emotion Terms Mixed Model Results**

| Language dimension | Fixed effects | *B* | | *SE* | | *df* | | *t* | | | *p* | | |
| --- | --- | --- | --- | --- | --- | --- | --- | --- | --- | --- | --- | --- | --- |
| Negative emotion terms (%) | Intercept | 1.43E+00 | 1.22E-02 | | 2.74E+03 | | 117.336 | | < .001 | | |  |  |
|  | Gender: Men | -2.30E-02 | 4.87E-03 | | 3.72E+04 | | -4.729 | | < .001 | | |  |  |
|  | Ethnicity: Other | -1.80E-02 | 7.11E-03 | | 4.48E+04 | | -2.540 | | .011 | | |  |  |
|  | Ethnicity: Asian | -6.07E-02 | 1.41E-02 | | 4.22E+04 | | -4.291 | | < .001 | | |  |  |
|  | Ethnicity: Black/African | -3.79E-02 | 8.63E-03 | | 3.02E+04 | | -4.387 | | < .001 | | |  |  |
|  | Ethnicity: Hispanic or Latino | 1.08E-03 | 1.30E-02 | | 3.55E+04 | | 0.083 | | .934 | | |  |  |
|  | Random effects | *n* | | σ^2^ | | *SD* |  | | |  | | |  |
|  | Hospitalization ID | 58,361 | | 0.12291 | | 0.3506 |  | | |  | | |  |
|  | Subject ID | 46,139 | | 0.04348 | | 0.2085 |  | | |  | | |  |
|  | Diagnosis | 15,589 | | 0.05048 | | 0.2247 |  | | |  | | |  |
|  | Caregiver ID | 1,913 | | 0.19844 | | 0.4455 |  | | |  | | |  |
|  | *R*^2^m | 0.0001647139 | |  | |  |  | | |  | | |  |
|  | *R*^2^c | 0.1977454 | |  | |  |  | | |  | | |  |

*Note*. The reference group for the gender fixed effect is “Women” and the reference group for the ethnicity fixed effect is “White.” *R^2^m* refers to the marginal *R^2^*, which accounts for variance explained by the fixed effects in linear mixed model calculations (gender and ethnicity). *R^2^c* refers to the conditional *R^2^*, which accounts for variance explained by the fixed and random effects in linear mixed model calculations.

These results suggest physicians use more negative emotion terms when attending to women compared to men after controlling for the patient’s ethnicity (and the random effects as well). Physicians attending to Black/African and Asian patients focus on fewer negative emotion terms compared to White patients, after controlling for patient’s gender (and the random effects as well). Please refer to Supplementary Table S8 for *Bonferroni*-corrected multiple comparisons across ethnicity categories.

# **Supplementary Table S4: Body Terms Mixed Model Results**

| Language dimension | Fixed effects | *B* | *SE* | *df* | *t* | *p* |
| --- | --- | --- | --- | --- | --- | --- |
| Body terms (%) | Intercept | 1.85E+00 | 3.00E-02 | 2.15E+03 | 61.699 | < .001 |
|  | Gender: Men | 3.96E-02 | 6.32E-03 | 3.28E+04 | 6.276 | < .001 |
|  | Ethnicity: Other | 2.19E-02 | 9.19E-03 | 3.92E+04 | 2.388 | .017 |
|  | Ethnicity: Asian | 9.64E-02 | 1.82E-02 | 3.60E+04 | 5.309 | < .001 |
|  | Ethnicity: Black/African | -8.07E-02 | 1.12E-02 | 2.78E+04 | -7.200 | < .001 |
|  | Ethnicity: Hispanic or Latino | 3.81E-02 | 1.68E-02 | 3.12E+04 | 2.272 | .023 |
|  | Random effects | *n* | σ^2^ | *SD* |  |  |
|  | Hospitalization ID | 58,361 | 0.1297 | 0.3601 |  |  |
|  | Subject ID | 46,139 | 0.1032 | 0.3212 |  |  |
|  | Diagnosis | 15,589 | 0.2639 | 0.5138 |  |  |
|  | Caregiver ID | 1,913 | 1.501 | 1.2252 |  |  |
|  | *R*^2^m | 0.0003006192 |  |  |  |  |
|  | *R*^2^c | 0.400258 |  |  |  |  |

*Note*. The reference group for the gender fixed effect is “Women” and the reference group for the ethnicity fixed effect is “White.” *R^2^m* refers to the marginal *R^2^*, which accounts for variance explained by the fixed effects in linear mixed model calculations (gender and ethnicity). *R^2^c* refers to the conditional *R^2^*, which accounts for variance explained by the fixed and random effects in linear mixed model calculations.

These results suggest physicians use more body terms when attending to men compared to women after controlling for the patient’s ethnicity (and the random effects as well). Physicians attending to Black/African patients focus on fewer body terms compared to physicians attending to White patients, after controlling for patient’s gender (and the random effects as well). However, physicians attending to Asian and Hispanic or Latino patients focus on more body terms compared to physicians attending to White patients, after controlling for patient’s gender (and the random effects as well).

The results, taken together with Table S2 and S3, suggest by focusing more on the emotions of certain patients (women compared to men), physicians also fail to adequately focus on their bodily diagnoses and biological elements of their care.

Please refer to Supplementary Table S8 for *Bonferroni*-corrected multiple comparisons across ethnicity categories.

# **Supplementary Table S5: Impersonal Pronouns Mixed Model Results**

| Language dimension | Fixed effects | *B* | *SE* | *df* | *t* | *p* |
| --- | --- | --- | --- | --- | --- | --- |
| Impersonal pronouns (%) | Intercept | 7.05E-01 | 1.35E-02 | 1.85E+03 | 52.146 | < .001 |
|  | Gender: Men | -5.26E-03 | 2.24E-03 | 2.83E+04 | -2.352 | .019 |
|  | Ethnicity: Other | 1.23E-02 | 3.29E-03 | 3.49E+04 | 3.724 | < .001 |
|  | Ethnicity: Asian | -2.98E-03 | 6.60E-03 | 3.03E+04 | -0.452 | .652 |
|  | Ethnicity: Black/African | 3.47E-03 | 3.93E-03 | 2.01E+04 | 0.882 | .378 |
|  | Ethnicity: Hispanic or Latino | -2.90E-03 | 5.96E-03 | 2.64E+04 | -0.486 | .627 |
|  | Random effects | *n* | σ^2^ | *SD* |  |  |
|  | Hospitalization ID | 58,361 | 0.014376 | 0.1199 |  |  |
|  | Subject ID | 46,139 | 0.003738 | 0.06114 |  |  |
|  | Diagnosis | 15,589 | 0.007995 | 0.08941 |  |  |
|  | Caregiver ID | 1,913 | 0.312041 | 0.55861 |  |  |
|  | *R*^2^m | 2.197928e-05 |  |  |  |  |
|  | *R*^2^c | 0.2813872 |  |  |  |  |

*Note*. The reference group for the gender fixed effect is “Women” and the reference group for the ethnicity fixed effect is “White.” *R^2^m* refers to the marginal *R^2^*, which accounts for variance explained by the fixed effects in linear mixed model calculations (gender and ethnicity). *R^2^c* refers to the conditional *R^2^*, which accounts for variance explained by the fixed and random effects in linear mixed model calculations.

These results suggest physicians use more impersonal pronouns when attending to women compared to men after controlling for the patient’s ethnicity (and the random effects as well).

# **Supplementary Table S6: Analytic Thinking Mixed Model Results**

| Language dimension | Fixed effects | *B* | *SE* | *df* | *t* | *p* |
| --- | --- | --- | --- | --- | --- | --- |
| Analytic thinking | Intercept | 9.29E+01 | 8.02E-02 | 1.91E+03 | 1157.97 | < .001 |
|  | Gender: Men | 8.00E-02 | 1.35E-02 | 2.75E+04 | 5.935 | < .001 |
|  | Ethnicity: Other | -4.91E-02 | 1.98E-02 | 3.34E+04 | -2.484 | .013 |
|  | Ethnicity: Asian | -5.32E-02 | 3.99E-02 | 2.99E+04 | -1.334 | .182 |
|  | Ethnicity: Black/African | -1.37E-01 | 2.39E-02 | 2.06E+04 | -5.733 | < .001 |
|  | Ethnicity: Hispanic or Latino | -8.47E-02 | 3.61E-02 | 2.59E+04 | -2.344 | .019 |
|  | Random effects | *n* | σ^2^ | *SD* |  |  |
|  | Hospitalization ID | 58,361 | 0.6194 | 0.787 |  |  |
|  | Subject ID | 46,139 | 0.1933 | 0.4396 |  |  |
|  | Diagnosis | 15,589 | 0.1206 | 0.3472 |  |  |
|  | Caregiver ID | 1,913 | 11.0727 | 3.3276 |  |  |
|  | *R*^2^m | 9.311905e-05 |  |  |  |  |
|  | *R*^2^c | 0.3050829 |  |  |  |  |

*Note*. The reference group for the gender fixed effect is “Women” and the reference group for the ethnicity fixed effect is “White.” *R^2^m* refers to the marginal *R^2^*, which accounts for variance explained by the fixed effects in linear mixed model calculations (gender and ethnicity). *R^2^c* refers to the conditional *R^2^*, which accounts for variance explained by the fixed and random effects in linear mixed model calculations.

These results suggest physicians think in a more analytic and reasoned manner when attending to men compared to women after controlling for the patient’s ethnicity (and the random effects as well). Physicians attending to Black/African patients think in a less analytic, more dynamic and storytelling manner compared to physicians attending to White patients, after controlling for patient’s gender (and the random effects as well). Please refer to Supplementary Table S8 for *Bonferroni*-corrected multiple comparisons across ethnicity categories.

# **Supplementary Table S7: Cognitive Processes Mixed Model Results**

| Language dimension | Fixed effects | *B* | *SE* | *df* | *t* | *p* |
| --- | --- | --- | --- | --- | --- | --- |
| Cognitive processes (%) | Intercept | 4.655E+00 | 3.038E-02 | 2.304E+03 | 153.235 | < .001 |
|  | Gender: Men | -4.36E-02 | 9.95E-03 | 3.02E+04 | -4.380 | < .001 |
|  | Ethnicity: Other | -1.76E-01 | 1.45E-02 | 3.57E+04 | -12.155 | < .001 |
|  | Ethnicity: Asian | 1.147E-01 | 2.915E-02 | 3.342E+04 | 3.935 | < .001 |
|  | Ethnicity: Black/African | 1.230E-01 | 1.768E-02 | 2.479E+04 | 6.957 | < .001 |
|  | Ethnicity: Hispanic or Latino | 1.508E-02 | 2.664E-02 | 2.884E+04 | 0.566 | .571 |
|  | Random effects | *n* | σ^2^ | *SD* |  |  |
|  | Hospitalization ID | 58,361 | 0.424 | 0.6512 |  |  |
|  | Subject ID | 46,139 | 0.1714 | 0.414 |  |  |
|  | Diagnosis | 15,589 | 0.1268 | 0.3561 |  |  |
|  | Caregiver ID | 1,913 | 1.3467 | 1.1605 |  |  |
|  | *R*^2^m | 0.0005640454 |  |  |  |  |
|  | *R*^2^c | 0.1764386 |  |  |  |  |

*Note*. The reference group for the gender fixed effect is “Women” and the reference group for the ethnicity fixed effect is “White.” *R^2^m* refers to the marginal *R^2^*, which accounts for variance explained by the fixed effects in linear mixed model calculations (gender and ethnicity). *R^2^c* refers to the conditional *R^2^*, which accounts for variance explained by the fixed and random effects in linear mixed model calculations.

These results suggest physicians use fewer cognitive processing words when attending to men compared to women after controlling for the patient’s ethnicity (and the random effects as well), evidence of needing to “work through” diagnoses of women more than men. Physicians attending to Black/African and Asian patients use more cognitive processing terms compared to physicians attending to White patients, after controlling for patient’s gender (and the random effects as well), evidence of needing to “work through” and put in more cognitive effort to understand diagnoses of certain non-White patients more than White patients. Please refer to Supplementary Table S8 for *Bonferroni*-corrected multiple comparisons across ethnicity categories.

# **Further Interrogation of Impersonal Pronouns and Gender Finding**

An alternative explanation for the relationship between impersonal pronouns and gender is that impersonal references might be linked to diagnoses (e.g., “*it* needs to be removed”) or the situation (e.g., “*it* is unclear what to do next.”), and not only a gender bias. To evaluate how impersonal pronouns might be used differently according to patient gender, a dictionary of 8 “she” words was created from the LIWC2015 *shehe* variable (the words include *her*, *hers*, *herself*, *she*, *she’d*, *she’ll*, *she’s*, *shes*). In a linear mixed model — predicting rates of impersonal pronouns from ethnicity, “she” words, and other controls such as the subject ID, admission ID, diagnosis, and caregiver ID — the data suggest impersonal pronouns and she words were positively related (*B* = 1.398e-01, *SE* = 1.255e-03, *t* = 111.44, *p* < .001, *R*^2^*c* = 0.225). Therefore, after controlling for the patient’s ethnicity (and other relevant controls for data non-independence), the more that physicians focus on women, the more that they attend to the patient and their healthcare experience in impersonal terms.

Does this pattern also hold for direct men references? A “he” word dictionary was created from 8 words in the LIWC2015 *shehe* category as well (words include *he*, *he’d*, *he’s*, *hes*, *him*, *himself*, *his*, *hissel**). The relationship between “he” words and impersonal pronouns was also statistically significant and positive in a linear mixed model predicting rates of impersonal pronouns from ethnicity, “he” words, and other controls such as the subject ID, admission ID, diagnosis, and caregiver ID (*B* = 1.299e-01, *SE* = 1.318e-03, *t* = 98.59, *p* < .001, *R*^2^*c* = 0.230).

Given these relationships, a qualitative review of caregiver notes was performed and revealed that impersonal pronouns referred to multiple aspects of the patient experience: direct references to people (e.g., “**This** unfortunate 44 yr old woman returns”), in addition to diagnoses (e.g., “BELIEVES **THAT IT** IS PROBABLY MUSCULAR”) and the situation (e.g., “Obviously **something** is bothering her but declining offers of emotional support”). Words in bold are impersonal pronouns and it is clear that impersonal pronouns are often accompanied by direct women references (e.g., “she” and “her” words) more than direct men references (“he” words). Therefore, one interpretation of these data suggests when attending to women, physicians focus on their overall healthcare experience (e.g., the patient, their diagnosis, the situation) with more impersonality than when attending to men. Greater rates of impersonality suggest an increase in psychological distancing throughout the healthcare experience when physicians attend to women compared to men.

Finally, to investigate if the strength of the prior relationships differed across genders, simple bivariate correlations were conducted between impersonal pronouns and “she” words, *r*(1851279) = .127, *p* < .001, and impersonal pronouns and “he” words, *r*(1851279) = .104, *p* < .001. Fisher’s *z*-transformations suggested the magnitude of these correlations were significantly different from each other (*z* = 22.18, *p* < .001, two-tailed). Therefore, while an increase in impersonal pronouns may be associated with an increase in men and women references throughout one’s healthcare experience (e.g., the patient, their diagnosis, the situation), this effect is significantly stronger when a physician focuses on women references.

Together, impersonal pronouns are not a substitute for direct women or men references (e.g., when impersonal pronouns increase, “she” or “he” words do not directly decrease). Instead, they are used in tandem and with multiple purposes to reveal a disparity in what physicians focus on when they attend to patients of different genders.

# **Supplementary Table S8: Linguistic Mean Differences Across Ethnicities**

| Positive emotion terms (%) | Estimate | *SE* | *df* | *t* | *p* | Impersonal pronouns (%) | Estimate | *SE* | *df* | *t* | *p* |
| --- | --- | --- | --- | --- | --- | --- | --- | --- | --- | --- | --- |
| Asian - Black | -0.004 | 0.013 | 30824 | -0.33 | 1.000 | Asian - Black | -0.006 | 0.007 | 27517 | -0.87 | 1.000 |
| Asian - Hispanic or Latino | -0.039 | 0.015 | 31894 | -2.59 | .095 | Asian - Hispanic or Latino | 0.000 | 0.009 | 28513 | -0.01 | 1.000 |
| Asian - Other | -0.059 | 0.012 | 35740 | -4.74 | < .001 | Asian - Other | -0.015 | 0.007 | 31398 | -2.14 | .327 |
| Asian - White | -0.040 | 0.012 | 34132 | -3.51 | .004 | Asian - White | -0.003 | 0.007 | 30302 | -0.45 | 1.000 |
| Black - Hispanic or Latino | -0.035 | 0.012 | 26924 | -2.90 | .037 | Black - Hispanic or Latino | 0.006 | 0.007 | 23971 | 0.93 | 1.000 |
| Black - Other | -0.055 | 0.008 | 29133 | -6.52 | < .001 | Black - Other | -0.009 | 0.005 | 26030 | -1.83 | .672 |
| Black - White | -0.036 | 0.007 | 22755 | -5.21 | < .001 | Black - White | 0.003 | 0.004 | 20105 | 0.88 | 1.000 |
| Hispanic or Latino - Other | -0.019 | 0.011 | 31856 | -1.70 | .887 | Hispanic or Latino - Other | -0.015 | 0.007 | 28536 | -2.31 | .208 |
| Hispanic or Latino - White | -0.001 | 0.010 | 29265 | -0.10 | 1.000 | Hispanic or Latino - White | -0.003 | 0.006 | 26418 | -0.49 | 1.000 |
| Other - White | 0.018 | 0.006 | 40441 | 3.26 | .011 | Other - White | 0.012 | 0.003 | 34938 | 3.72 | .002 |
| Negative emotion terms (%) | Estimate | *SE* | *df* | *t* | *p* | Analytic thinking | Estimate | SE | *df* | *t* | *p* |
| Asian - Black | -0.023 | 0.016 | 39031 | -1.42 | 1.000 | Asian - Black | 0.084 | 0.045 | 27411 | 1.86 | .634 |
| Asian - Hispanic or Latino | -0.062 | 0.019 | 39166 | -3.29 | .010 | Asian - Hispanic or Latino | 0.031 | 0.053 | 28087 | 0.60 | 1.000 |
| Asian - Other | -0.043 | 0.015 | 43369 | -2.79 | .054 | Asian - Other | -0.004 | 0.043 | 30891 | -0.10 | 1.000 |
| Asian - White | -0.061 | 0.014 | 42230 | -4.29 | < .001 | Asian - White | -0.053 | 0.040 | 29909 | -1.33 | 1.000 |
| Black - Hispanic or Latino | -0.039 | 0.015 | 33511 | -2.59 | .096 | Black - Hispanic or Latino | -0.052 | 0.042 | 23928 | -1.25 | 1.000 |
| Black - Other | -0.020 | 0.010 | 36833 | -1.89 | .588 | Black - Other | -0.088 | 0.029 | 25965 | -3.02 | .025 |
| Black - White | -0.038 | 0.009 | 30224 | -4.39 | < .001 | Black - White | -0.137 | 0.024 | 20610 | -5.73 | < .001 |
| Hispanic or Latino - Other | 0.019 | 0.014 | 37959 | 1.34 | 1.000 | Hispanic or Latino - Other | -0.036 | 0.040 | 27841 | -0.90 | 1.000 |
| Hispanic or Latino - White | 0.001 | 0.013 | 35543 | 0.08 | 1.000 | Hispanic or Latino - White | -0.085 | 0.036 | 25929 | -2.34 | .191 |
| Other - White | -0.018 | 0.007 | 44799 | -2.54 | .111 | Other - White | -0.049 | 0.020 | 33383 | -2.48 | .130 |
| Body terms (%) | Estimate | *SE* | *df* | *t* | *p* | Cognitive processes (%) | Estimate | *SE* | *df* | *t* | *p* |
| Asian - Black | 0.177 | 0.021 | 33755 | 8.59 | < .001 | Asian - Black | -0.008 | 0.033 | 31161 | -0.25 | 1.000 |
| Asian - Hispanic or Latino | 0.058 | 0.024 | 33763 | 2.42 | .156 | Asian - Hispanic or Latino | 0.100 | 0.039 | 31320 | 2.59 | .098 |
| Asian - Other | 0.075 | 0.020 | 36976 | 3.79 | .002 | Asian - Other | 0.291 | 0.032 | 34282 | 9.24 | < .001 |
| Asian - White | 0.096 | 0.018 | 35984 | 5.31 | < .001 | Asian - White | 0.115 | 0.029 | 33418 | 3.94 | .001 |
| Black - Hispanic or Latino | -0.119 | 0.019 | 29810 | -6.12 | < .001 | Black - Hispanic or Latino | 0.108 | 0.031 | 27287 | 3.50 | .005 |
| Black - Other | -0.103 | 0.014 | 32772 | -7.55 | < .001 | Black - Other | 0.299 | 0.021 | 29764 | 13.95 | < .001 |
| Black - White | -0.081 | 0.011 | 27803 | -7.20 | < .001 | Black - White | 0.123 | 0.018 | 24793 | 6.96 | < .001 |
| Hispanic or Latino - Other | 0.016 | 0.018 | 33105 | 0.88 | 1.000 | Hispanic or Latino - Other | 0.191 | 0.029 | 30628 | 6.55 | < .001 |
| Hispanic or Latino - White | 0.038 | 0.017 | 31200 | 2.27 | .231 | Hispanic or Latino - White | 0.015 | 0.027 | 28835 | 0.57 | 1.000 |
| Other - White | 0.022 | 0.009 | 39174 | 2.39 | .170 | Other - White | -0.176 | 0.015 | 35647 | -12.16 | < .001 |

*Note*. *p-*values are *Bonferroni*-corrected. Mean differences are results after including controls reported in the main text.

# **Supplementary Table S9: Frequency of Content Words by Gender**

|  | Men | | | Women | | |
| --- | --- | --- | --- | --- | --- | --- |
| Number | Word | Frequency | % | Word | Frequency | % |
| 1 | pt | 2,889,443 | 39.23 | pt | 1,245,080 | 39.11 |
| 2 | mg | 1,915,629 | 15.19 | mg | 842,352 | 15.33 |
| 3 | name | 1,914,635 | 39.18 | name | 826,336 | 38.85 |
| 4 | ml | 1,867,565 | 11.98 | ml | 804,296 | 12.06 |
| 5 | left | 1,746,954 | 31.83 | left | 735,792 | 30.84 |
| 6 | right | 1,590,800 | 30.20 | right | 673,833 | 29.26 |
| 7 | patient | 1,445,596 | 23.82 | patient | 623,165 | 23.56 |
| 8 | pm | 1,292,010 | 22.35 | pm | 542,745 | 21.81 |
| 9 | plan | 1,248,208 | 38.27 | plan | 541,806 | 38.36 |
| 10 | chest | 1,105,836 | 26.63 | po | 475,494 | 21.10 |
| 11 | hr | 1,079,189 | 31.56 | hr | 473,509 | 32.01 |
| 12 | pain | 1,063,945 | 21.32 | pain | 472,615 | 21.31 |
| 13 | dl | 1,059,892 | 8.04 | dl | 456,619 | 8.00 |
| 14 | po | 1,050,611 | 20.51 | chest | 447,419 | 25.40 |
| 15 | normal | 1,001,680 | 20.16 | normal | 437,674 | 20.12 |
| 16 | continue | 994,311 | 24.12 | continue | 437,199 | 24.42 |
| 17 | blood | 972,570 | 19.36 | blood | 426,814 | 19.24 |
| 18 | assessment | 947,722 | 23.70 | assessment | 404,241 | 23.44 |
| 19 | day | 911,085 | 20.67 | day | 404,128 | 20.85 |
| 20 | reason | 884,661 | 22.69 | stable | 383,779 | 29.47 |
| 21 | status | 879,250 | 29.37 | status | 382,082 | 29.11 |
| 22 | stable | 876,639 | 29.14 | reason | 364,145 | 21.49 |
| 23 | noted | 818,034 | 26.38 | history | 359,122 | 20.03 |
| 24 | clip | 815,020 | 20.26 | noted | 355,280 | 26.39 |
| 25 | history | 814,041 | 20.00 | hospital | 338,044 | 29.12 |
| 26 | tube | 800,053 | 19.97 | clip | 335,654 | 19.12 |
| 27 | hospital | 789,177 | 30.04 | respiratory | 326,923 | 22.39 |
| 28 | ct | 777,320 | 16.77 | cont | 326,805 | 17.73 |
| 29 | respiratory | 744,597 | 22.16 | care | 326,679 | 28.20 |
| 30 | iv | 744,238 | 20.88 | iv | 326,394 | 20.93 |
| 31 | cont | 740,735 | 17.58 | ct | 325,955 | 16.22 |
| 32 | care | 738,722 | 27.87 | tube | 321,354 | 18.79 |
| 33 | monitor | 717,271 | 23.16 | monitor | 313,511 | 23.41 |
| 34 | clear | 665,040 | 28.07 | bp | 294,013 | 24.82 |
| 35 | bp | 658,268 | 24.36 | clear | 291,659 | 28.38 |
| 36 | medical | 648,842 | 30.40 | medical | 276,627 | 29.59 |
| 37 | acute | 637,743 | 18.01 | tablet | 272,034 | 2.13 |
| 38 | examination | 627,422 | 28.75 | acute | 271,451 | 17.89 |
| 39 | fluid | 621,778 | 18.27 | fluid | 268,693 | 18.33 |
| 40 | tablet | 610,758 | 2.09 | kg | 265,194 | 18.27 |
| 41 | meq | 605,142 | 8.73 | examination | 262,870 | 27.71 |
| 42 | remains | 604,271 | 22.21 | remains | 261,658 | 22.28 |
| 43 | kg | 601,756 | 18.02 | soft | 261,571 | 27.35 |
| 44 | response | 601,568 | 15.15 | meq | 261,051 | 8.69 |
| 45 | soft | 589,508 | 26.71 | response | 259,334 | 15.03 |
| 46 | daily | 588,135 | 8.72 | daily | 259,241 | 8.64 |
| 47 | radiology | 584,455 | 28.07 | today | 252,180 | 19.60 |
| 48 | 24 | 584,240 | 14.82 | rr | 250,989 | 22.34 |
| 49 | today | 579,174 | 19.36 | 24 | 250,508 | 14.95 |
| 50 | sounds | 569,330 | 18.26 | support | 249,091 | 22.87 |

# **Supplementary Table S10: Frequency of Content Words by Ethnicity**

|  | Asian | | | Black/African | | | Hispanic or Latino | | |
| --- | --- | --- | --- | --- | --- | --- | --- | --- | --- |
| Number | Word | Frequency | % | Word | Frequency | % | Word | Frequency | % |
| 1 | pt | 61,594 | 29.79 | pt | 274,257 | 37.64 | pt | 85,290 | 33.67 |
| 2 | name | 53,731 | 37.64 | mg | 197,389 | 15.49 | name | 67,424 | 38.62 |
| 3 | ml | 49,953 | 10.98 | name | 187,411 | 37.74 | mg | 62,571 | 13.77 |
| 4 | mg | 44,947 | 12.06 | ml | 185,167 | 12.84 | ml | 59,728 | 11.39 |
| 5 | left | 42,304 | 26.10 | left | 145,686 | 28.22 | left | 56,526 | 29.32 |
| 6 | right | 39,468 | 25.03 | patient | 143,881 | 22.62 | right | 54,153 | 28.30 |
| 7 | patient | 35,573 | 19.63 | right | 134,552 | 26.31 | patient | 44,948 | 22.02 |
| 8 | pm | 35,045 | 19.96 | plan | 126,581 | 39.12 | pm | 41,159 | 22.19 |
| 9 | plan | 32,810 | 35.35 | pm | 119,456 | 20.89 | plan | 40,169 | 36.22 |
| 10 | continue | 31,570 | 25.30 | po | 114,653 | 21.86 | pain | 39,778 | 20.64 |
| 11 | normal | 29,740 | 20.59 | continue | 111,216 | 26.28 | normal | 37,629 | 21.17 |
| 12 | stable | 29,449 | 33.45 | blood | 110,298 | 20.60 | po | 37,436 | 20.73 |
| 13 | blood | 29,208 | 19.14 | pain | 106,110 | 19.77 | chest | 36,771 | 25.07 |
| 14 | infant | 28,698 | 19.44 | hr | 105,460 | 32.46 | continue | 34,433 | 24.24 |
| 15 | dl | 28,226 | 7.14 | dl | 105,128 | 8.36 | blood | 34,413 | 19.40 |
| 16 | chest | 27,696 | 22.47 | day | 97,950 | 21.62 | dl | 33,774 | 7.40 |
| 17 | day | 27,564 | 22.76 | normal | 96,658 | 20.40 | day | 33,136 | 21.35 |
| 18 | po | 27,297 | 20.75 | assessment | 93,431 | 23.85 | hr | 32,976 | 29.00 |
| 19 | hr | 26,800 | 28.02 | chest | 89,740 | 22.99 | stable | 32,878 | 30.66 |
| 20 | cont | 25,153 | 18.33 | status | 88,287 | 28.73 | reason | 31,788 | 22.89 |
| 21 | noted | 24,231 | 26.24 | stable | 87,182 | 30.11 | clip | 29,697 | 20.51 |
| 22 | monitor | 23,966 | 25.49 | history | 87,155 | 20.33 | assessment | 29,569 | 22.03 |
| 23 | reason | 23,189 | 19.79 | noted | 82,784 | 27.44 | ct | 28,628 | 16.57 |
| 24 | assessment | 22,979 | 20.20 | monitor | 77,640 | 25.01 | history | 28,136 | 19.37 |
| 25 | kg | 22,374 | 22.72 | cont | 77,147 | 18.08 | noted | 27,907 | 25.60 |
| 26 | status | 21,540 | 25.72 | respiratory | 76,703 | 23.60 | status | 27,487 | 27.63 |
| 27 | clear | 21,478 | 31.60 | care | 74,895 | 28.73 | hospital | 27,073 | 29.43 |
| 28 | history | 21,385 | 18.72 | bp | 72,391 | 25.81 | cont | 26,611 | 17.80 |
| 29 | clip | 21,240 | 17.60 | iv | 68,786 | 19.67 | monitor | 25,804 | 23.67 |
| 30 | pain | 20,786 | 14.73 | reason | 68,470 | 18.06 | tube | 24,563 | 17.18 |
| 31 | soft | 20,472 | 30.66 | hospital | 68,457 | 25.35 | iv | 24,388 | 19.92 |
| 32 | respiratory | 20,423 | 20.45 | tablet | 68,009 | 2.32 | clear | 24,259 | 29.46 |
| 33 | ct | 20,297 | 14.46 | kg | 67,279 | 20.24 | respiratory | 23,415 | 19.66 |
| 34 | tube | 20,267 | 16.86 | clear | 67,046 | 29.07 | kg | 23,393 | 19.50 |
| 35 | care | 20,262 | 25.00 | renal | 65,778 | 13.98 | care | 23,118 | 25.44 |
| 36 | support | 19,359 | 24.23 | ct | 64,902 | 14.47 | soft | 22,892 | 28.90 |
| 37 | family | 19,271 | 20.76 | acute | 64,381 | 18.13 | infant | 22,569 | 13.31 |
| 38 | hospital | 19,116 | 25.20 | soft | 63,892 | 29.04 | tablet | 22,419 | 2.23 |
| 39 | rr | 18,376 | 24.06 | today | 63,422 | 21.30 | medical | 21,985 | 29.58 |
| 40 | remains | 18,171 | 23.05 | daily | 63,366 | 8.91 | examination | 21,916 | 28.56 |
| 41 | sounds | 17,321 | 19.35 | remains | 62,754 | 23.26 | bp | 21,845 | 22.59 |
| 42 | note | 17,260 | 27.21 | clip | 62,566 | 15.79 | remains | 21,306 | 22.54 |
| 43 | bp | 17,253 | 22.28 | infant | 62,207 | 13.61 | contrast | 21,054 | 9.22 |
| 44 | medical | 17,070 | 26.79 | tube | 61,677 | 16.36 | radiology | 20,733 | 27.83 |
| 45 | iv | 17,060 | 16.53 | admission | 61,183 | 14.57 | family | 20,040 | 19.87 |
| 46 | 24 | 16,920 | 15.60 | rr | 60,983 | 24.65 | fluid | 20,034 | 16.75 |
| 47 | examination | 16,748 | 25.54 | failure | 60,883 | 15.55 | support | 19,962 | 22.50 |
| 48 | abd | 16,594 | 25.50 | response | 60,827 | 14.75 | 24 | 19,850 | 14.89 |
| 49 | feeds | 16,360 | 17.42 | 24 | 60,596 | 16.78 | daily | 19,685 | 8.08 |
| 50 | today | 16,347 | 18.82 | meq | 60,270 | 8.77 | acute | 19,574 | 15.99 |

**Supplementary Table S10: Frequency of Content Words by Ethnicity (continued)**

|  | Other | | | White | | |
| --- | --- | --- | --- | --- | --- | --- |
| Number | Word | Frequency | % | Word | Frequency | % |
| 1 | pt | 348,653 | 35.49 | pt | 2,119,677 | 40.81 |
| 2 | name | 231,481 | 35.20 | mg | 1,434,497 | 16.09 |
| 3 | left | 222,448 | 29.20 | ml | 1,421,122 | 12.82 |
| 4 | right | 196,712 | 27.32 | name | 1,374,589 | 40.20 |
| 5 | mg | 176,225 | 11.32 | left | 1,279,991 | 33.18 |
| 6 | patient | 170,914 | 19.55 | right | 1,165,915 | 31.58 |
| 7 | ml | 151,595 | 7.42 | patient | 1,050,293 | 25.06 |
| 8 | chest | 145,333 | 24.84 | pm | 969,744 | 23.33 |
| 9 | hr | 136,698 | 28.71 | plan | 916,668 | 39.53 |
| 10 | plan | 131,995 | 32.15 | dl | 807,876 | 8.74 |
| 11 | pm | 126,609 | 18.83 | chest | 806,296 | 27.70 |
| 12 | normal | 122,395 | 18.19 | pain | 796,316 | 22.66 |
| 13 | stable | 121,138 | 29.65 | hr | 777,261 | 32.23 |
| 14 | reason | 118,832 | 22.29 | po | 768,286 | 21.07 |
| 15 | day | 116,108 | 19.87 | assessment | 719,916 | 25.56 |
| 16 | continue | 115,188 | 21.34 | normal | 715,408 | 20.43 |
| 17 | clip | 112,903 | 20.49 | continue | 701,906 | 24.27 |
| 18 | tube | 109,834 | 19.82 | blood | 695,500 | 19.90 |
| 19 | status | 105,816 | 25.78 | reason | 642,382 | 23.50 |
| 20 | noted | 104,849 | 24.36 | day | 636,330 | 20.54 |
| 21 | cont | 103,243 | 17.39 | status | 636,123 | 30.37 |
| 22 | blood | 103,153 | 15.61 | stable | 605,994 | 28.61 |
| 23 | po | 102,943 | 16.44 | history | 594,168 | 20.87 |
| 24 | hospital | 102,170 | 28.44 | clip | 588,614 | 20.91 |
| 25 | pain | 100,956 | 17.00 | tube | 583,714 | 20.74 |
| 26 | monitor | 92,739 | 21.96 | noted | 578,265 | 26.63 |
| 27 | ct | 91,579 | 14.35 | hospital | 572,361 | 31.19 |
| 28 | care | 90,418 | 25.01 | ct | 571,917 | 17.64 |
| 29 | remains | 90,408 | 23.85 | iv | 553,134 | 21.96 |
| 30 | clear | 87,833 | 27.89 | respiratory | 548,288 | 22.99 |
| 31 | kg | 85,365 | 18.42 | care | 530,095 | 28.52 |
| 32 | resp | 84,968 | 26.16 | cont | 508,582 | 17.48 |
| 33 | dl | 84,888 | 4.60 | monitor | 497,125 | 22.98 |
| 34 | history | 83,197 | 15.67 | acute | 476,783 | 19.23 |
| 35 | soft | 82,397 | 26.94 | bp | 472,782 | 24.91 |
| 36 | assessment | 81,899 | 15.20 | medical | 472,332 | 31.60 |
| 37 | iv | 80,870 | 17.29 | clear | 464,424 | 27.70 |
| 38 | examination | 79,560 | 25.85 | fluid | 461,226 | 19.25 |
| 39 | medical | 77,327 | 27.00 | meq | 460,613 | 9.44 |
| 40 | infant | 76,264 | 11.74 | tablet | 457,590 | 2.20 |
| 41 | respiratory | 75,804 | 17.76 | response | 456,450 | 16.37 |
| 42 | support | 75,161 | 20.95 | examination | 456,274 | 29.96 |
| 43 | bp | 73,998 | 21.23 | daily | 439,812 | 9.25 |
| 44 | today | 72,990 | 18.08 | 24 | 428,965 | 15.10 |
| 45 | number | 72,955 | 23.18 | radiology | 428,027 | 29.36 |
| 46 | note | 71,728 | 24.98 | remains | 411,636 | 21.65 |
| 47 | radiology | 71,367 | 24.96 | icu | 409,077 | 12.15 |
| 48 | report | 70,637 | 23.67 | today | 407,204 | 19.35 |
| 49 | small | 69,563 | 17.61 | sounds | 406,774 | 18.27 |
| 50 | sounds | 69,441 | 17.38 | kg | 403,347 | 17.32 |

*Note*. Percentages in Supplementary Tables S9 and S10 refer to the percentage of cases (caregiver notes) that contained the word in question. Words in Supplementary Tables S9 and S10 are arranged in terms of raw frequency of occurrence.

# **Supplementary Table S11: Means Across Groups for Interaction Effects**

| Language dimension | Ethnicity | Gender | *M* | *SE* | 95% CI |
| --- | --- | --- | --- | --- | --- |
| Positive emotion (%) | Asian | Women | 2.13 | 0.037 | [2.06, 2.20] |
|  | Black/African | Women | 2.10 | 0.034 | [2.04, 2.17] |
|  | Hispanic or Latino | Women | 2.16 | 0.036 | [2.09, 2.23] |
|  | Other | Women | 2.19 | 0.033 | [2.12, 2.25] |
|  | White | Women | 2.17 | 0.033 | [2.10, 2.23] |
|  | Asian | Men | 2.10 | 0.036 | [2.03, 2.17] |
|  | Black/African | Men | 2.14 | 0.034 | [2.07, 2.20] |
|  | Hispanic or Latino | Men | 2.15 | 0.035 | [2.08, 2.21] |
|  | Other | Men | 2.16 | 0.033 | [2.10, 2.23] |
|  | White | Men | 2.14 | 0.033 | [2.08, 2.21] |
|  |  |  |  |  |  |
| Negative emotion (%) | Asian | Women | 1.37 | 0.024 | [1.33, 1.42] |
|  | Black/African | Women | 1.40 | 0.016 | [1.36, 1.43] |
|  | Hispanic or Latino | Women | 1.43 | 0.023 | [1.38, 1.48] |
|  | Other | Women | 1.41 | 0.015 | [1.38, 1.44] |
|  | White | Women | 1.44 | 0.012 | [1.41, 1.46] |
|  | Asian | Men | 1.35 | 0.022 | [1.31, 1.39] |
|  | Black/African | Men | 1.38 | 0.017 | [1.34, 1.41] |
|  | Hispanic or Latino | Men | 1.42 | 0.020 | [1.38, 1.46] |
|  | Other | Men | 1.40 | 0.014 | [1.37, 1.42] |
|  | White | Men | 1.41 | 0.012 | [1.39, 1.43] |
|  |  |  |  |  |  |
| Body terms (%) | Asian | Women | 1.97 | 0.040 | [1.89, 2.05] |
|  | Black/African | Women | 1.75 | 0.033 | [1.69, 1.82] |
|  | Hispanic or Latino | Women | 1.87 | 0.040 | [1.80, 1.95] |
|  | Other | Women | 1.86 | 0.032 | [1.80, 1.92] |
|  | White | Women | 1.85 | 0.030 | [1.79, 1.91] |
|  | Asian | Men | 1.97 | 0.038 | [1.89, 2.04] |
|  | Black/African | Men | 1.82 | 0.033 | [1.76, 1.89] |
|  | Hispanic or Latino | Men | 1.94 | 0.036 | [1.86, 2.01] |
|  | Other | Men | 1.92 | 0.032 | [1.86, 1.98] |
|  | White | Men | 1.89 | 0.030 | [1.83, 1.94] |

# **Supplementary Table S11: Means Across Groups for Interaction Effects (Continued)**

| Language dimension | Ethnicity | Gender | *M* | *SE* | 95% CI |
| --- | --- | --- | --- | --- | --- |
| Impersonal pronouns (%) | Asian | Women | 0.690 | 0.017 | [0.66, 0.72] |
|  | Black/African | Women | 0.701 | 0.014 | [0.67, 0.73] |
|  | Hispanic or Latino | Women | 0.709 | 0.016 | [0.68, 0.74] |
|  | Other | Women | 0.718 | 0.014 | [0.69, 0.75] |
|  | White | Women | 0.706 | 0.014 | [0.68, 0.73] |
|  | Asian | Men | 0.705 | 0.016 | [0.67, 0.74] |
|  | Black/African | Men | 0.712 | 0.015 | [0.68, 0.74] |
|  | Hispanic or Latino | Men | 0.693 | 0.015 | [0.66, 0.72] |
|  | Other | Men | 0.711 | 0.014 | [0.68, 0.74] |
|  | White | Men | 0.699 | 0.014 | [0.67, 0.73] |
|  |  |  |  |  |  |
| Analytic thinking | Asian | Women | 92.81 | 0.100 | [92.61, 93.00] |
|  | Black/African | Women | 92.74 | 0.085 | [92.58, 92.91] |
|  | Hispanic or Latino | Women | 92.78 | 0.098 | [92.58, 92.97] |
|  | Other | Women | 92.82 | 0.084 | [92.65, 92.98] |
|  | White | Women | 92.86 | 0.080 | [92.70, 93.02] |
|  | Asian | Men | 92.89 | 0.094 | [92.71, 93.08] |
|  | Black/African | Men | 92.78 | 0.086 | [92.62, 92.95] |
|  | Hispanic or Latino | Men | 92.86 | 0.091 | [92.68, 93.04] |
|  | Other | Men | 92.89 | 0.083 | [92.73, 93.05] |
|  | White | Men | 92.95 | 0.080 | [92.79, 93.10] |
|  |  |  |  |  |  |
| Cognitive processes (%) | Asian | Women | 4.71 | 0.053 | [4.61, 4.82] |
|  | Black/African | Women | 4.81 | 0.037 | [4.74, 4.88] |
|  | Hispanic or Latino | Women | 4.62 | 0.051 | [4.52, 4.72] |
|  | Other | Women | 4.49 | 0.036 | [4.42, 4.56] |
|  | White | Women | 4.65 | 0.031 | [4.59, 4.71] |
|  | Asian | Men | 4.77 | 0.048 | [4.67, 4.86] |
|  | Black/African | Men | 4.69 | 0.038 | [4.62, 4.77] |
|  | Hispanic or Latino | Men | 4.66 | 0.044 | [4.57, 4.75] |
|  | Other | Men | 4.43 | 0.034 | [4.36, 4.49] |
|  | White | Men | 4.61 | 0.030 | [4.55, 4.67] |

# **Supplementary Table S12: Interaction Effect Mean Differences for Positive Emotion Terms**

| Language dimension | Group 1 | Group 2 | Estimate | SE | *z* | *p* |
| --- | --- | --- | --- | --- | --- | --- |
| Positive emotion terms (%) | Asian Women | Black/African Women | 0.028 | 0.020 | 1.45 | 1.000 |
|  | Asian Women | Hispanic or Latino Women | -0.029 | 0.024 | -1.22 | 1.000 |
|  | Asian Women | Other Women | -0.054 | 0.019 | -2.81 | .225 |
|  | Asian Women | White Women | -0.033 | 0.018 | -1.86 | 1.000 |
|  | Asian Women | Asian Men | 0.034 | 0.023 | 1.47 | 1.000 |
|  | Asian Women | Black/African Men | -0.004 | 0.020 | -0.22 | 1.000 |
|  | Asian Women | Hispanic or Latino Men | -0.013 | 0.022 | -0.59 | 1.000 |
|  | Asian Women | Other Men | -0.029 | 0.019 | -1.52 | 1.000 |
|  | Asian Women | White Men | -0.012 | 0.018 | -0.66 | 1.000 |
|  | Black/African Women | Hispanic or Latino Women | -0.058 | 0.019 | -3.10 | .087 |
|  | **Black/African Women** | **Other Women** | **-0.082** | **0.012** | **-6.91** | **< .001** |
|  | **Black/African Women** | **White Women** | **-0.062** | **0.010** | **-6.46** | **< .001** |
|  | Black/African Women | Asian Men | 0.005 | 0.017 | 0.30 | 1.000 |
|  | Black/African Women | Black/African Men | -0.033 | 0.013 | -2.51 | .548 |
|  | Black/African Women | Hispanic or Latino Men | -0.041 | 0.016 | -2.62 | .394 |
|  | **Black/African Women** | **Other Men** | **-0.057** | **0.011** | **-5.09** | **< .001** |
|  | **Black/African Women** | **White Men** | **-0.040** | **0.009** | **-4.27** | **.001** |
|  | Hispanic or Latino Women | Other Women | -0.025 | 0.018 | -1.37 | 1.000 |
|  | Hispanic or Latino Women | White Women | -0.004 | 0.017 | -0.25 | 1.000 |
|  | Hispanic or Latino Women | Asian Men | 0.063 | 0.022 | 2.85 | .194 |
|  | Hispanic or Latino Women | Black/African Men | 0.025 | 0.019 | 1.31 | 1.000 |
|  | Hispanic or Latino Women | Hispanic or Latino Men | 0.016 | 0.021 | 0.78 | 1.000 |
|  | Hispanic or Latino Women | Other Men | 0.001 | 0.018 | 0.03 | 1.000 |
|  | Hispanic or Latino Women | White Men | 0.017 | 0.017 | 1.05 | 1.000 |
|  | Other Women | White Women | 0.021 | 0.009 | 2.40 | .740 |
|  | **Other Women** | **Asian Men** | **0.088** | **0.017** | **5.24** | **< .001** |
|  | **Other Women** | **Black/African Men** | **0.050** | **0.012** | **3.99** | **.003** |
|  | Other Women | Hispanic or Latino Men | 0.041 | 0.015 | 2.69 | .322 |
|  | Other Women | Other Men | 0.025 | 0.010 | 2.47 | .616 |
|  | **Other Women** | **White Men** | **0.042** | **0.008** | **4.98** | **< .001** |
|  | **White Women** | **Asian Men** | **0.067** | **0.015** | **4.42** | **< .001** |
|  | White Women | Black/African Men | 0.029 | 0.010 | 2.83 | .207 |
|  | White Women | Hispanic or Latino Men | 0.020 | 0.014 | 1.50 | 1.000 |
|  | White Women | Other Men | 0.005 | 0.008 | 0.62 | 1.000 |
|  | **White Women** | **White Men** | **0.022** | **0.005** | **4.67** | **< .001** |
|  | Asian Men | Black/African Men | -0.038 | 0.018 | -2.16 | 1.000 |
|  | Asian Men | Hispanic or Latino Men | -0.046 | 0.020 | -2.37 | .808 |
|  | **Asian Men** | **Other Men** | **-0.062** | **0.016** | **-3.84** | **.006** |
|  | Asian Men | White Men | -0.045 | 0.015 | -3.02 | .115 |
|  | Black/African Men | Hispanic or Latino Men | -0.009 | 0.016 | -0.53 | 1.000 |
|  | Black/African Men | Other Men | -0.024 | 0.012 | -2.07 | 1.000 |
|  | Black/African Men | White Men | -0.007 | 0.010 | -0.74 | 1.000 |
|  | Hispanic or Latino Men | Other Men | -0.016 | 0.015 | -1.06 | 1.000 |
|  | Hispanic or Latino Men | White Men | 0.001 | 0.013 | 0.09 | 1.000 |
|  | Other Men | White Men | 0.017 | 0.007 | 2.29 | .997 |

*Note*. Significance values are *Bonferroni*-corrected. Bolded significance values represent *p* < .05.

# **Supplementary Table S13: Interaction Effect Mean Differences for Negative Emotion Terms**

| Language dimension | Group 1 | Group 2 | Estimate | SE | *z* | *p* |
| --- | --- | --- | --- | --- | --- | --- |
| Negative emotion terms (%) | Asian Women | Black/African Women | -0.023 | 0.024 | -0.96 | 1.000 |
|  | Asian Women | Hispanic or Latino Women | -0.057 | 0.029 | -1.93 | 1.000 |
|  | Asian Women | Other Women | -0.041 | 0.024 | -1.76 | 1.000 |
|  | Asian Women | White Women | -0.063 | 0.022 | -2.90 | .170 |
|  | Asian Women | Asian Men | 0.021 | 0.028 | 0.75 | 1.000 |
|  | Asian Women | Black/African Men | -0.002 | 0.024 | -0.10 | 1.000 |
|  | Asian Women | Hispanic or Latino Men | -0.044 | 0.027 | -1.65 | 1.000 |
|  | Asian Women | Other Men | -0.022 | 0.023 | -0.98 | 1.000 |
|  | Asian Women | White Men | -0.038 | 0.022 | -1.76 | 1.000 |
|  | Black/African Women | Hispanic or Latino Women | -0.034 | 0.023 | -1.46 | 1.000 |
|  | Black/African Women | Other Women | -0.019 | 0.015 | -1.24 | 1.000 |
|  | **Black/African Women** | **White Women** | **-0.040** | **0.012** | **-3.38** | **.033** |
|  | Black/African Women | Asian Men | 0.044 | 0.021 | 2.06 | 1.000 |
|  | Black/African Women | Black/African Men | 0.020 | 0.016 | 1.27 | 1.000 |
|  | Black/African Women | Hispanic or Latino Men | -0.021 | 0.020 | -1.08 | 1.000 |
|  | Black/African Women | Other Men | 0.000 | 0.014 | 0.03 | 1.000 |
|  | Black/African Women | White Men | -0.015 | 0.012 | -1.31 | 1.000 |
|  | Hispanic or Latino Women | Other Women | 0.015 | 0.023 | 0.67 | 1.000 |
|  | Hispanic or Latino Women | White Women | -0.006 | 0.021 | -0.30 | 1.000 |
|  | Hispanic or Latino Women | Asian Men | 0.078 | 0.027 | 2.85 | .197 |
|  | Hispanic or Latino Women | Black/African Men | 0.054 | 0.024 | 2.30 | .954 |
|  | Hispanic or Latino Women | Hispanic or Latino Men | 0.013 | 0.026 | 0.48 | 1.000 |
|  | Hispanic or Latino Women | Other Men | 0.034 | 0.022 | 1.55 | 1.000 |
|  | Hispanic or Latino Women | White Men | 0.018 | 0.021 | 0.89 | 1.000 |
|  | Other Women | White Women | -0.021 | 0.011 | -1.96 | 1.000 |
|  | Other Women | Asian Men | 0.062 | 0.021 | 3.01 | .118 |
|  | Other Women | Black/African Men | 0.039 | 0.016 | 2.50 | .555 |
|  | Other Women | Hispanic or Latino Men | -0.003 | 0.019 | -0.14 | 1.000 |
|  | Other Women | Other Men | 0.019 | 0.013 | 1.46 | 1.000 |
|  | Other Women | White Men | 0.003 | 0.011 | 0.31 | 1.000 |
|  | **White Women** | **Asian Men** | **0.084** | **0.019** | **4.50** | **< .001** |
|  | **White Women** | **Black/African Men** | **0.060** | **0.013** | **4.77** | **< .001** |
|  | White Women | Hispanic or Latino Men | 0.019 | 0.017 | 1.12 | 1.000 |
|  | **White Women** | **Other Men** | **0.040** | **0.010** | **4.25** | **.001** |
|  | **White Women** | **White Men** | **0.025** | **0.006** | **4.29** | **.001** |
|  | Asian Men | Black/African Men | -0.023 | 0.022 | -1.08 | 1.000 |
|  | Asian Men | Hispanic or Latino Men | -0.065 | 0.024 | -2.67 | .339 |
|  | Asian Men | Other Men | -0.043 | 0.020 | -2.17 | 1.000 |
|  | Asian Men | White Men | -0.059 | 0.019 | -3.19 | .064 |
|  | Black/African Men | Hispanic or Latino Men | -0.042 | 0.020 | -2.08 | 1.000 |
|  | Black/African Men | Other Men | -0.020 | 0.015 | -1.38 | 1.000 |
|  | Black/African Men | White Men | -0.036 | 0.012 | -2.86 | .190 |
|  | Hispanic or Latino Men | Other Men | 0.022 | 0.018 | 1.18 | 1.000 |
|  | Hispanic or Latino Men | White Men | 0.006 | 0.017 | 0.35 | 1.000 |
|  | Other Men | White Men | -0.016 | 0.009 | -1.70 | 1.000 |

*Note*. Significance values are *Bonferroni*-corrected. Bolded significance values represent *p* < .05.

# **Supplementary Table S14: Interaction Effect Mean Differences for Body Terms**

| Language dimension | Group 1 | Group 2 | Estimate | SE | *z* | *p* |
| --- | --- | --- | --- | --- | --- | --- |
| Body terms (%) | **Asian Women** | **Black/African Women** | **0.215** | **0.031** | **6.96** | **< .001** |
|  | Asian Women | Hispanic or Latino Women | 0.094 | 0.038 | 2.49 | .570 |
|  | **Asian Women** | **Other Women** | **0.108** | **0.030** | **3.56** | **.017** |
|  | **Asian Women** | **White Women** | **0.115** | **0.028** | **4.13** | **.002** |
|  | Asian Women | Asian Men | 0.000 | 0.036 | -0.01 | 1.000 |
|  | **Asian Women** | **Black/African Men** | **0.143** | **0.031** | **4.57** | **< .001** |
|  | Asian Women | Hispanic or Latino Men | 0.033 | 0.034 | 0.96 | 1.000 |
|  | Asian Women | Other Men | 0.051 | 0.029 | 1.72 | 1.000 |
|  | Asian Women | White Men | 0.083 | 0.028 | 2.97 | .136 |
|  | **Black/African Women** | **Hispanic or Latino Women** | **-0.120** | **0.030** | **-4.05** | **.002** |
|  | **Black/African Women** | **Other Women** | **-0.107** | **0.019** | **-5.53** | **< .001** |
|  | **Black/African Women** | **White Women** | **-0.099** | **0.015** | **-6.44** | **< .001** |
|  | **Black/African Women** | **Asian Men** | **-0.215** | **0.027** | **-7.88** | **< .001** |
|  | **Black/African Women** | **Black/African Men** | **-0.071** | **0.021** | **-3.41** | **.029** |
|  | **Black/African Women** | **Hispanic or Latino Men** | **-0.182** | **0.025** | **-7.17** | **< .001** |
|  | **Black/African Women** | **Other Men** | **-0.164** | **0.018** | **-9.06** | **< .001** |
|  | **Black/African Women** | **White Men** | **-0.132** | **0.015** | **-8.69** | **< .001** |
|  | Hispanic or Latino Women | Other Women | 0.013 | 0.029 | 0.46 | 1.000 |
|  | Hispanic or Latino Women | White Women | 0.021 | 0.027 | 0.79 | 1.000 |
|  | Hispanic or Latino Women | Asian Men | -0.095 | 0.035 | -2.71 | .304 |
|  | Hispanic or Latino Women | Black/African Men | 0.049 | 0.030 | 1.62 | 1.000 |
|  | Hispanic or Latino Women | Hispanic or Latino Men | -0.061 | 0.033 | -1.83 | 1.000 |
|  | Hispanic or Latino Women | Other Men | -0.044 | 0.028 | -1.54 | 1.000 |
|  | Hispanic or Latino Women | White Men | -0.012 | 0.027 | -0.44 | 1.000 |
|  | Other Women | White Women | 0.008 | 0.014 | 0.56 | 1.000 |
|  | **Other Women** | **Asian Men** | **-0.108** | **0.027** | **-4.06** | **.002** |
|  | Other Women | Black/African Men | 0.036 | 0.020 | 1.77 | 1.000 |
|  | Other Women | Hispanic or Latino Men | -0.075 | 0.025 | -3.03 | .111 |
|  | **Other Women** | **Other Men** | **-0.057** | **0.017** | **-3.41** | **.029** |
|  | Other Women | White Men | -0.025 | 0.014 | -1.81 | 1.000 |
|  | **White Women** | **Asian Men** | **-0.116** | **0.024** | **-4.85** | **< .001** |
|  | White Women | Black/African Men | 0.028 | 0.016 | 1.70 | 1.000 |
|  | **White Women** | **Hispanic or Latino Men** | **-0.082** | **0.022** | **-3.80** | **.006** |
|  | **White Women** | **Other Men** | **-0.065** | **0.012** | **-5.28** | **< .001** |
|  | **White Women** | **White Men** | **-0.033** | **0.007** | **-4.41** | **.001** |
|  | **Asian Men** | **Black/African Men** | **0.144** | **0.028** | **5.17** | **< .001** |
|  | Asian Men | Hispanic or Latino Men | 0.033 | 0.031 | 1.07 | 1.000 |
|  | Asian Men | Other Men | 0.051 | 0.026 | 1.99 | 1.000 |
|  | **Asian Men** | **White Men** | **0.083** | **0.024** | **3.50** | **.021** |
|  | **Black/African Men** | **Hispanic or Latino Men** | **-0.110** | **0.026** | **-4.26** | **.001** |
|  | **Black/African Men** | **Other Men** | **-0.093** | **0.019** | **-4.91** | **< .001** |
|  | **Black/African Men** | **White Men** | **-0.061** | **0.016** | **-3.76** | **.008** |
|  | Hispanic or Latino Men | Other Men | 0.018 | 0.024 | 0.75 | 1.000 |
|  | Hispanic or Latino Men | White Men | 0.049 | 0.021 | 2.30 | .955 |
|  | Other Men | White Men | 0.032 | 0.012 | 2.69 | .321 |

*Note*. Significance values are *Bonferroni*-corrected. Bolded significance values represent *p* < .05.

# **Supplementary Table S15: Interaction Effect Mean Differences for Impersonal Pronouns**

| Language dimension | Group 1 | Group 2 | Estimate | SE | *z* | *p* |
| --- | --- | --- | --- | --- | --- | --- |
| Impersonal pronouns (%) | Asian Women | Black/African Women | -0.011 | 0.011 | -0.95 | 1.000 |
|  | Asian Women | Hispanic or Latino Women | -0.019 | 0.014 | -1.37 | 1.000 |
|  | Asian Women | Other Women | -0.028 | 0.011 | -2.53 | .511 |
|  | Asian Women | White Women | -0.016 | 0.010 | -1.55 | 1.000 |
|  | Asian Women | Asian Men | -0.015 | 0.013 | -1.13 | 1.000 |
|  | Asian Women | Black/African Men | -0.022 | 0.011 | -1.92 | 1.000 |
|  | Asian Women | Hispanic or Latino Men | -0.002 | 0.013 | -0.18 | 1.000 |
|  | Asian Women | Other Men | -0.021 | 0.011 | -1.95 | 1.000 |
|  | Asian Women | White Men | -0.009 | 0.010 | -0.85 | 1.000 |
|  | Black/African Women | Hispanic or Latino Women | -0.008 | 0.011 | -0.76 | 1.000 |
|  | Black/African Women | Other Women | -0.017 | 0.007 | -2.54 | .501 |
|  | Black/African Women | White Women | -0.005 | 0.005 | -0.98 | 1.000 |
|  | Black/African Women | Asian Men | -0.004 | 0.010 | -0.42 | 1.000 |
|  | Black/African Women | Black/African Men | -0.011 | 0.007 | -1.53 | 1.000 |
|  | Black/African Women | Hispanic or Latino Men | 0.008 | 0.009 | 0.94 | 1.000 |
|  | Black/African Women | Other Men | -0.010 | 0.006 | -1.62 | 1.000 |
|  | Black/African Women | White Men | 0.002 | 0.005 | 0.39 | 1.000 |
|  | Hispanic or Latino Women | Other Women | -0.009 | 0.010 | -0.89 | 1.000 |
|  | Hispanic or Latino Women | White Women | 0.003 | 0.010 | 0.29 | 1.000 |
|  | Hispanic or Latino Women | Asian Men | 0.004 | 0.013 | 0.32 | 1.000 |
|  | Hispanic or Latino Women | Black/African Men | -0.003 | 0.011 | -0.29 | 1.000 |
|  | Hispanic or Latino Women | Hispanic or Latino Men | 0.016 | 0.012 | 1.38 | 1.000 |
|  | Hispanic or Latino Women | Other Men | -0.002 | 0.010 | -0.23 | 1.000 |
|  | Hispanic or Latino Women | White Men | 0.010 | 0.009 | 1.07 | 1.000 |
|  | Other Women | White Women | 0.012 | 0.005 | 2.40 | .743 |
|  | Other Women | Asian Men | 0.013 | 0.010 | 1.38 | 1.000 |
|  | Other Women | Black/African Men | 0.006 | 0.007 | 0.86 | 1.000 |
|  | Other Women | Hispanic or Latino Men | 0.026 | 0.009 | 2.94 | .149 |
|  | Other Women | Other Men | 0.007 | 0.006 | 1.16 | 1.000 |
|  | **Other Women** | **White Men** | **0.019** | **0.005** | **3.93** | **.004** |
|  | White Women | Asian Men | 0.001 | 0.009 | 0.14 | 1.000 |
|  | White Women | Black/African Men | -0.006 | 0.006 | -1.03 | 1.000 |
|  | White Women | Hispanic or Latino Men | 0.014 | 0.008 | 1.77 | 1.000 |
|  | White Women | Other Men | -0.005 | 0.004 | -1.16 | 1.000 |
|  | White Women | White Men | 0.007 | 0.003 | 2.77 | .255 |
|  | Asian Men | Black/African Men | -0.007 | 0.010 | -0.71 | 1.000 |
|  | Asian Men | Hispanic or Latino Men | 0.012 | 0.011 | 1.12 | 1.000 |
|  | Asian Men | Other Men | -0.006 | 0.009 | -0.68 | 1.000 |
|  | Asian Men | White Men | 0.006 | 0.009 | 0.72 | 1.000 |
|  | Black/African Men | Hispanic or Latino Men | 0.020 | 0.009 | 2.14 | 1.000 |
|  | Black/African Men | Other Men | 0.001 | 0.007 | 0.13 | 1.000 |
|  | Black/African Men | White Men | 0.013 | 0.006 | 2.33 | .896 |
|  | Hispanic or Latino Men | Other Men | -0.019 | 0.008 | -2.23 | 1.000 |
|  | Hispanic or Latino Men | White Men | -0.006 | 0.008 | -0.83 | 1.000 |
|  | Other Men | White Men | 0.012 | 0.004 | 2.91 | .165 |

*Note*. Significance values are *Bonferroni*-corrected. Bolded significance values represent *p* < .05.

# **Supplementary Table S16: Interaction Effect Mean Differences for Analytic Thinking**

| Language dimension | Group 1 | Group 2 | Estimate | SE | *z* | *p* |
| --- | --- | --- | --- | --- | --- | --- |
| Analytic thinking | Asian Women | Black/African Women | 0.061 | 0.068 | 0.90 | 1.000 |
|  | Asian Women | Hispanic or Latino Women | 0.030 | 0.083 | 0.36 | 1.000 |
|  | Asian Women | Other Women | -0.012 | 0.067 | -0.18 | 1.000 |
|  | Asian Women | White Women | -0.055 | 0.062 | -0.88 | 1.000 |
|  | Asian Women | Asian Men | -0.088 | 0.079 | -1.11 | 1.000 |
|  | Asian Women | Black/African Men | 0.020 | 0.069 | 0.29 | 1.000 |
|  | Asian Women | Hispanic or Latino Men | -0.055 | 0.076 | -0.73 | 1.000 |
|  | Asian Women | Other Men | -0.086 | 0.065 | -1.32 | 1.000 |
|  | Asian Women | White Men | -0.140 | 0.062 | -2.27 | 1.000 |
|  | Black/African Women | Hispanic or Latino Women | -0.031 | 0.064 | -0.49 | 1.000 |
|  | Black/African Women | Other Women | -0.073 | 0.041 | -1.77 | 1.000 |
|  | **Black/African Women** | **White Women** | **-0.116** | **0.033** | **-3.54** | **.018** |
|  | Black/African Women | Asian Men | -0.149 | 0.059 | -2.51 | .544 |
|  | Black/African Women | Black/African Men | -0.041 | 0.045 | -0.92 | 1.000 |
|  | Black/African Women | Hispanic or Latino Men | -0.116 | 0.054 | -2.14 | 1.000 |
|  | **Black/African Women** | **Other Men** | **-0.147** | **0.039** | **-3.81** | **.006** |
|  | **Black/African Women** | **White Men** | **-0.201** | **0.032** | **-6.24** | **< .001** |
|  | Hispanic or Latino Women | Other Women | -0.042 | 0.063 | -0.67 | 1.000 |
|  | Hispanic or Latino Women | White Women | -0.085 | 0.058 | -1.46 | 1.000 |
|  | Hispanic or Latino Women | Asian Men | -0.118 | 0.076 | -1.55 | 1.000 |
|  | Hispanic or Latino Women | Black/African Men | -0.010 | 0.065 | -0.15 | 1.000 |
|  | Hispanic or Latino Women | Hispanic or Latino Men | -0.085 | 0.072 | -1.18 | 1.000 |
|  | Hispanic or Latino Women | Other Men | -0.116 | 0.061 | -1.90 | 1.000 |
|  | Hispanic or Latino Women | White Men | -0.170 | 0.058 | -2.96 | .139 |
|  | Other Women | White Women | -0.042 | 0.030 | -1.40 | 1.000 |
|  | Other Women | Asian Men | -0.075 | 0.058 | -1.30 | 1.000 |
|  | Other Women | Black/African Men | 0.032 | 0.043 | 0.75 | 1.000 |
|  | Other Women | Hispanic or Latino Men | -0.043 | 0.053 | -0.81 | 1.000 |
|  | Other Women | Other Men | -0.074 | 0.036 | -2.05 | 1.000 |
|  | **Other Women** | **White Men** | **-0.128** | **0.030** | **-4.31** | **.001** |
|  | White Women | Asian Men | -0.033 | 0.052 | -0.63 | 1.000 |
|  | White Women | Black/African Men | 0.075 | 0.035 | 2.13 | 1.000 |
|  | White Women | Hispanic or Latino Men | 0.000 | 0.047 | -0.01 | 1.000 |
|  | White Women | Other Men | -0.032 | 0.027 | -1.19 | 1.000 |
|  | **White Women** | **White Men** | **-0.086** | **0.016** | **-5.37** | **< .001** |
|  | Asian Men | Black/African Men | 0.108 | 0.061 | 1.78 | 1.000 |
|  | Asian Men | Hispanic or Latino Men | 0.033 | 0.068 | 0.48 | 1.000 |
|  | Asian Men | Other Men | 0.001 | 0.056 | 0.03 | 1.000 |
|  | Asian Men | White Men | -0.053 | 0.052 | -1.01 | 1.000 |
|  | Black/African Men | Hispanic or Latino Men | -0.075 | 0.056 | -1.35 | 1.000 |
|  | Black/African Men | Other Men | -0.106 | 0.041 | -2.62 | .396 |
|  | **Black/African Men** | **White Men** | **-0.160** | **0.035** | **-4.63** | **< .001** |
|  | Hispanic or Latino Men | Other Men | -0.031 | 0.051 | -0.62 | 1.000 |
|  | Hispanic or Latino Men | White Men | -0.085 | 0.046 | -1.84 | 1.000 |
|  | Other Men | White Men | -0.054 | 0.026 | -2.10 | 1.000 |

*Note*. Significance values are *Bonferroni*-corrected. Bolded significance values represent *p* < .05.

# **Supplementary Table S17: Interaction Effect Mean Differences for Cognitive Processes**

| Language dimension | Group 1 | Group 2 | Estimate | SE | *z* | *p* |
| --- | --- | --- | --- | --- | --- | --- |
| Cognitive processes (%) | Asian Women | Black/African Women | -0.101 | 0.049 | -2.04 | 1.000 |
|  | Asian Women | Hispanic or Latino Women | 0.095 | 0.061 | 1.57 | 1.000 |
|  | **Asian Women** | **Other Women** | **0.220** | **0.049** | **4.54** | **< .001** |
|  | Asian Women | White Women | 0.060 | 0.045 | 1.34 | 1.000 |
|  | Asian Women | Asian Men | -0.055 | 0.058 | -0.96 | 1.000 |
|  | Asian Women | Black/African Men | 0.018 | 0.050 | 0.36 | 1.000 |
|  | Asian Women | Hispanic or Latino Men | 0.052 | 0.055 | 0.94 | 1.000 |
|  | **Asian Women** | **Other Men** | **0.286** | **0.047** | **6.05** | **< .001** |
|  | Asian Women | White Men | 0.098 | 0.045 | 2.20 | 1.000 |
|  | **Black/African Women** | **Hispanic or Latino Women** | **0.196** | **0.047** | **4.14** | **.002** |
|  | **Black/African Women** | **Other Women** | **0.321** | **0.031** | **10.51** | **< .001** |
|  | **Black/African Women** | **White Women** | **0.161** | **0.024** | **6.63** | **< .001** |
|  | Black/African Women | Asian Men | 0.046 | 0.044 | 1.05 | 1.000 |
|  | **Black/African Women** | **Black/African Men** | **0.119** | **0.033** | **3.59** | **.015** |
|  | **Black/African Women** | **Hispanic or Latino Men** | **0.153** | **0.040** | **3.80** | **.007** |
|  | **Black/African Women** | **Other Men** | **0.387** | **0.029** | **13.54** | **< .001** |
|  | **Black/African Women** | **White Men** | **0.199** | **0.024** | **8.31** | **< .001** |
|  | Hispanic or Latino Women | Other Women | 0.125 | 0.046 | 2.70 | .309 |
|  | Hispanic or Latino Women | White Women | -0.035 | 0.043 | -0.82 | 1.000 |
|  | Hispanic or Latino Women | Asian Men | -0.150 | 0.056 | -2.69 | .324 |
|  | Hispanic or Latino Women | Black/African Men | -0.077 | 0.048 | -1.59 | 1.000 |
|  | Hispanic or Latino Women | Hispanic or Latino Men | -0.043 | 0.053 | -0.81 | 1.000 |
|  | **Hispanic or Latino Women** | **Other Men** | **0.191** | **0.045** | **4.24** | **.001** |
|  | Hispanic or Latino Women | White Men | 0.003 | 0.042 | 0.08 | 1.000 |
|  | **Other Women** | **White Women** | **-0.160** | **0.022** | **-7.19** | **< .001** |
|  | **Other Women** | **Asian Men** | **-0.276** | **0.043** | **-6.48** | **< .001** |
|  | **Other Women** | **Black/African Men** | **-0.202** | **0.032** | **-6.34** | **< .001** |
|  | **Other Women** | **Hispanic or Latino Men** | **-0.168** | **0.039** | **-4.31** | **.001** |
|  | Other Women | Other Men | 0.066 | 0.027 | 2.48 | .596 |
|  | **Other Women** | **White Men** | **-0.122** | **0.022** | **-5.58** | **< .001** |
|  | White Women | Asian Men | -0.115 | 0.038 | -3.01 | .119 |
|  | White Women | Black/African Men | -0.042 | 0.026 | -1.62 | 1.000 |
|  | White Women | Hispanic or Latino Men | -0.008 | 0.034 | -0.24 | 1.000 |
|  | **White Women** | **Other Men** | **0.226** | **0.019** | **11.62** | **< .001** |
|  | White Women | White Men | 0.038 | 0.012 | 3.24 | .054 |
|  | Asian Men | Black/African Men | 0.073 | 0.045 | 1.65 | 1.000 |
|  | Asian Men | Hispanic or Latino Men | 0.107 | 0.050 | 2.15 | 1.000 |
|  | **Asian Men** | **Other Men** | **0.341** | **0.041** | **8.30** | **< .001** |
|  | **Asian Men** | **White Men** | **0.153** | **0.038** | **4.03** | **.003** |
|  | Black/African Men | Hispanic or Latino Men | 0.034 | 0.041 | 0.82 | 1.000 |
|  | **Black/African Men** | **Other Men** | **0.268** | **0.030** | **8.95** | **< .001** |
|  | Black/African Men | White Men | 0.080 | 0.026 | 3.13 | .079 |
|  | **Hispanic or Latino Men** | **Other Men** | **0.234** | **0.037** | **6.26** | **< .001** |
|  | Hispanic or Latino Men | White Men | 0.046 | 0.034 | 1.36 | 1.000 |
|  | **Other Men** | **White Men** | **-0.188** | **0.019** | **-9.99** | **< .001** |

*Note*. Significance values are *Bonferroni*-corrected. Bolded significance values represent *p* < .05.
